# Use of a Smartphone-Based Mobile App for Weight Management in Obese Minority Stroke Survivors: Pilot Randomized Controlled Trial With Open Blinded End Point

**DOI:** 10.2196/17816

**Published:** 2020-04-22

**Authors:** Nneka L Ifejika, Minal Bhadane, Chunyan C Cai, Elizabeth A Noser, James C Grotta, Sean I Savitz

**Affiliations:** 1 Department of Physical Medicine and Rehabilitation University of Texas Southwestern Medical Center Dallas, TX United States; 2 Department of Neurology and Neurotherapeutics University of Texas Southwestern Medical Center Dallas, TX United States; 3 Department of Population and Data Sciences University of Texas Southwestern Medical Center Dallas, TX United States; 4 College of Optometry University of Houston Houston, TX United States; 5 Division of Clinical and Translational Sciences Department of Internal Medicine McGovern Medical School at the University of Texas Health Science Center at Houston Houston, TX United States; 6 Institute for Stroke and Cerebrovascular Disease University of Texas Health Science Center at Houston Houston, TX United States; 7 Department of Neurology McGovern Medical School at the University of Texas Health Science Center at Houston Houston, TX United States; 8 Clinical Innovation and Research Institute Memorial Hermann Hospital – Texas Medical Center Houston, TX United States

**Keywords:** smartphone, stroke, obesity, telemedicine, minority groups, cognitive dysfunction, outcome assessment, health care

## Abstract

**Background:**

Minorities have an increased incidence of early-onset, obesity-related cerebrovascular disease. Unfortunately, effective weight management in this vulnerable population has significant barriers.

**Objective:**

Our objective was to determine the feasibility and preliminary treatment effects of a smartphone-based weight loss intervention versus food journals to monitor dietary patterns in minority stroke patients.

**Methods:**

Swipe out Stroke was a pilot prospective randomized controlled trial with open blinded end point. Minority stroke patients and their caregivers were screened for participation using cluster enrollment. We used adaptive randomization for assignment to a behavior intervention with (1) smartphone-based self-monitoring or (2) food journal self-monitoring. The smartphone group used Lose it! to record meals and communicate with us. Reminder messages (first 30 days), weekly summaries plus reminder messages on missed days (days 31-90), and weekly summaries only (days 91-180) were sent via push notifications. The food journal group used paper diaries. Both groups received 4 in-person visits (baseline and 30, 90, and 180 days), culturally competent counseling, and educational materials. The primary outcome was reduced total body weight.

**Results:**

We enrolled 36 stroke patients (n=23, 64% African American; n=13, 36% Hispanic), 17 in the smartphone group, and 19 in the food journal group. Mean age was 54 (SD 9) years; mean body mass index was 35.7 (SD 5.7) kg/m2; education, employment status, and family history of stroke or obesity did not differ between the groups. Baseline rates of depression (Patient Health Questionnaire-9 [PHQ-9] score median 5.5, IQR 3.0-9.5), cognitive impairment (Montreal Cognitive Assessment score median 23.5, IQR 21-26), and inability to ambulate (5/36, 14% with modified Rankin Scale score 3) were similar. In total, 25 (69%) stroke survivors completed Swipe out Stroke (13/17 in the smartphone group, 12/19 in the food journal group); 1 participant in the smartphone group died. Median weight change at 180 days was 5.7 lb (IQR –2.4 to 8.0) in the smartphone group versus 6.4 lb (IQR –2.2 to 12.5; *P*=.77) in the food journal group. Depression was significantly lower at 30 days in the smartphone group than in the food journal group (PHQ-9 score 2 vs 8; *P*=.03). Clinically relevant depression rates remained in the zero to minimal range for the smartphone group compared with mild to moderate range in the food journal group at day 90 (PHQ-9 score 3.5 vs 4.5; *P*=.39) and day 180 (PHQ-9 score 3 vs 6; *P*=.12).

**Conclusions:**

In a population of obese minority stroke survivors, the use of a smartphone did not lead to a significant difference in weight change compared with keeping a food journal. The presence of baseline depression (19/36, 53%) was a confounding variable, which improved with app engagement. Future studies that include treatment of poststroke depression may positively influence intervention efficacy.

**Trial Registration:**

ClinicalTrials.gov NCT02531074; https://www.clinicaltrials.gov/ct2/show/NCT02531074

## Introduction

### Importance of the Problem

Decades of research have shown a disproportionate incidence of obesity among African American and Hispanic populations in the United States [1-3]. Minorities have a median age at first stroke, which is highly correlated with obesity, 10 to 13 years earlier than non-Hispanic whites [4,5]. The early onset of stroke in minorities is expensive. Direct medical costs for African American stroke patients are estimated to top US $16 billion in 2020; by 2030, stroke prevalence is expected to rise the most among Hispanic men, with direct costs of care increasing over 300% since 2012 [6].

Adherence to evidence-based therapies for obesity management in minority stroke patients is insufficient. Previous studies have demonstrated success through restrictive health promotion interventions [7] and stroke navigators [8], both of which require close follow-up by a medical professional. Unfortunately, inadequate finances and lack of health insurance are barriers to care in minorities [9]; therefore, study protocols that require frequent medical follow-up are not generalizable.

### Prior Work

The efficacy of smartphone-based self-monitoring to facilitate weight loss is well established. Wang et al [10] conducted a randomized controlled trial in a largely African American population, comparing a smartphone-based behavioral intervention for weight loss and glycemic control versus paper diaries or usual care. The smartphone group experienced a 2.73% weight loss at 6 months, compared with 0.13% weight loss in the paper diary group and a 0.49% weight gain in the usual-care group [10]. Minority populations are willing adopters of mobile health (mHealth) technology; a recent analysis by Asan et al of the Health Information National Trends Survey of American adults found that minorities were less likely to indicate they had no interest in exchanging health tips electronically with a health care provider [11].

However, there may be an attenuated effect of electronic device use in patients with chronic diseases, such as stroke. A study by Robbins et al showed that those with self-reported “very good or excellent” health or those who engaged in physical activity were more likely to download a health app [12]. Furthermore, in mHealth studies involving high-need, high-cost populations, Singh et al found that only 30.3% of the apps were identifiable and available to the public [13]. As stroke is a chronic end-organ disease with a high incidence of physical impairments, stroke survivors may have lower use of electronic interventions.

### Objectives

The Swipe out Stroke study tested the feasibility and preliminary treatment effects of using a readily available smartphone-based weight loss intervention in obese minority stroke patients versus food journals. Stroke affects the cognitive domains of attention, memory, language, and orientation [14]; approximately 30% of stroke patients develop dementia within 1 year [15]. These cognitive challenges may lead to limited mHealth research in the minority stroke population, which is increasing in volume and health care costs. We hypothesized that personal engagement with health care professionals through the mobile app would provide positive reinforcement and give support when participants experienced difficulty with program maintenance.

## Methods

### Study Design

Swipe out Stroke was a phase 1, pilot, prospective, randomized controlled trial with open blinded end point study. The blinded end point committee consisted of the senior authors (JG, SS) and the statistician (CC). We evaluated 2 groups—(1) a group receiving a behavior intervention with smartphone-based self-monitoring and (2) a group receiving a behavior intervention with food journal self-monitoring—for differences in weight loss at day 30, day 90, and day 180 of the study period. We used a mixed-methods design with quantitative measures. We distributed exit surveys to determine participants’ acceptance of the intervention; we report the quantitative results in this paper.

### Ethics

This study was approved by the Institutional Review Board at the McGovern Medical School at the University of Texas Health Science Center at Houston, Houston, Texas, USA (HSC-MS-13-0608). We obtained informed consent from both study participants and their caregivers, detailing the purpose of the study, frequency of visits, descriptors of the smartphone and food journal interventions, benefits, risks, protection of privacy, and study withdrawal procedures. The Consolidated Standards of Reporting Trials of Electronic and Mobile Health Applications and Online Telehealth (CONSORT-EHEALTH) checklist served as a guide for reporting this study (Multimedia Appendix 1 [16]). The trial is registered with ClinicalTrials.gov (NCT02531074).

### Patient Population and Setting

We screened obese African American or Hispanic patients age 18 years and older, acutely hospitalized for ischemic or hemorrhagic stroke. Textbox 1 outlines the inclusion and exclusion criteria. The setting was a Joint Commission–accredited Comprehensive Stroke Center in Houston, Texas, USA with a 322-km telemedicine radius, reaching areas with a large underinsured population. The Joint Commission is a US nonprofit organization that accredits more than 22,000 US health care organizations and programs. Joint Commission accreditation is recognized by the majority of US state governments as a condition of licensure for receiving Medicare and Medicaid reimbursements.

**Swipe out Stroke inclusion and exclusion criteria.**

**Inclusion criteria**

- Ischemic or hemorrhagic stroke.
- Age 18-85 years.
- African American or Hispanic ethnicity.
- Poststroke modified Rankin Scale (mRS) score 0-3.
- Poststroke body mass index >30 kg/m^2^.
- Prescription medication for diabetes mellitus, hypertension or hyperlipidemia.
- Willing to follow a healthy eating pattern and NOT use weight loss medications.
- Personal or caregiver ownership of a computer, smartphone or other smart device (iPhone or Android platform) with internet access.
- If patient has alexia, agraphia, acalculia, dementia or blindness, caregiver must be willing to complete the intervention.

**Exclusion criteria**

- Preexisting disability with mRS score ≥4.
- Contraindications to weight loss (planning to become pregnant, history of an eating disorder).
- Steroid use for suspected vasculitis.
- Current or recent (past 6 months) participation in a weight loss program or use of weight loss medication.

### Sample Size Estimate

We aimed to recruit 50 patients in Swipe out Stroke, with retention of 15 patients in each group (n=30). Based on clinical estimates, we anticipated a 50% completion rate in the smartphone group and a 10% completion rate in the food journal group. Due to several large regional flooding events during the study period (March 2015 to December 2016), we closed enrollment upon achieving 17 patients in the smartphone group and 19 patients in the food journal group (n=36). Completion rates in both groups were higher than clinical estimates.

### Randomization

We randomly assigned stroke survivors to either the smartphone group or food journal group in a 1 to 1 ratio, using an adaptive covariate randomization algorithm. The adaptive randomization schema used the Pearson chi-square statistic to measure treatment imbalances in stroke severity (modified Rankin Scale score of 0-1: no symptoms to no significant disability; or modified Rankin Scale score of 2-3: slight to moderate disability), age, and sex. We determined the presence of depression at randomization using the Patient Health Questionnaire-9 (PHQ-9) survey; we equally allocated participants with depression to the smartphone and food journal groups in 3 categories: zero to minimal (score 0-4), mild to moderate (5-14), and moderately severe to severe (>14). We recalculated treatment assignment probability for each new patient; random assignment using a Web-based management app achieved the best balance between the smartphone and food journal groups. Caregivers joined the group of the study participant to facilitate participation in the protocol and compliance with the study intervention.

### Intervention

#### Screening

Participants were screened by the stroke social worker and a member of the Swipe out Stroke research team during the acute hospitalization. After informed consent was obtained, the baseline clinic visit occurred within 2 weeks of hospital discharge.

#### Smartphone Group

During the baseline visit, a member of the study team created a user account for the mobile app (Lose It! version 5.2.1; FitNow, Inc, Boston, MA, USA) by using the participant’s email address. Each participant and caregiver (if applicable) received a tutorial on how to download and use Lose It! on his or her personally owned smartphone. Lose It! is a free mobile app.

**Figure 1:**
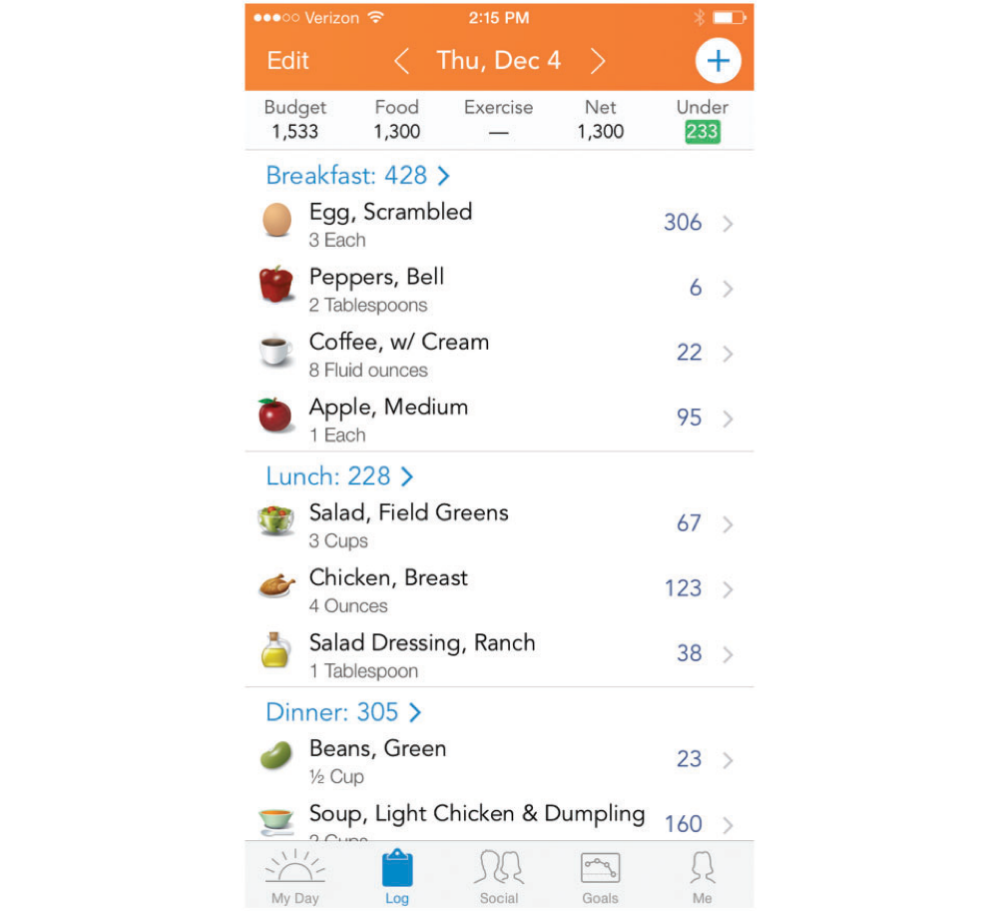
Example of a smartphone group Lose it! weight loss app screenshot.

For each participant, we implemented a 10% weight loss goal and provided a value for maximum daily caloric intake under the Budget column (Figure 1). Participants could search for foods, including restaurant items, or scan the bar codes of grocery items, with direct upload onto the Lose It! platform. Nutritional data were uploaded from food labels of grocery items as mandated by the US Food and Drug Administration since 1994, and from restaurants as mandated by each state. The number of calories consumed could be increased with physical activity and logged in the Exercise column, which varied based on exercise duration and intensity. The net calories each day equaled the total of food calories minus exercise calories (Figure 1).

During the initiation phase (baseline to day 30), we created a pattern of compliance. Participants received daily monitoring of caloric intake, with reminder messages via push notifications. During the application phase (day 31-90), weekly push notification summaries of compliance were sent, with reminder messages on missed days. The maintenance phase (days 91-180) consisted of weekly summaries with no reminder messages. Details of the smartphone intervention protocol and message examples have been published [17].

#### Food Journal Group

Similar to the smartphone group, for the food journal group we calculated a goal of 10% body weight reduction during the baseline visit. We used the pocket-sized *CalorieKing Food & Exercise Journal* [18]; each journal contained space to document 10 weeks of food intake; a weekly summary page; areas to record calories, fat, protein, and carbohydrate; and a 4-page mini-calorie, fat, and carbohydrate counter to reference beverages, meats, fruits, vegetables, cereals, snack foods, and popular fast food establishments.

#### Interventions for Both Groups

Participants in both groups received culturally competent dietary counseling by a clinician of their ethnic group, measuring cups, the US Department of Health and Human Services Dietary Approaches to Stop Hypertension eating plan guide, American Heart Association (AHA) cookbooks, and AHA reading materials (Suggested Servings from Each Food Group, Choosing a Restaurant, Dining Out Tips by Cuisine, and Ordering your Meal). At the end of each phase, both groups returned to the clinic for weight measurements and counseling. Study visits were at no cost to participants and caregivers.

### Feasibility and Treatment Fidelity

We measured study feasibility using retention rates at day 180 and adherence to the self-monitoring intervention at days 30, 90, and 180. We measured adherence to the smartphone intervention through a coaching tool integrated with the Lose It! platform (Ascend for Lose It!). Smartphone participants, with caregiver assistance if needed, were instructed to enter food daily, though either the functionality of the mobile app or free text. We measured adherence to the food journal intervention through review of written entries, with similar caregiver assistance.

### Measures

#### Demographics and Self-Reported Cerebrovascular Risk Factors

We collected data on participants’ age, sex, ethnicity, marital status, employment status, educational level, weight and height (to calculate body mass index), and stroke-related disability level in a sociodemographic questionnaire. We assessed cognitive impairments using the Montreal Cognitive Assessment (MoCA); a score of 26 and higher was considered normal [19]. We collected personal health, family history, and past medical history information using a general health history form.

#### Objective Cerebrovascular Risk Factors

We collected cerebrovascular risk factors in both groups, with normative ranges derived from AHA guidelines [20,21]. Baseline study measurements were collected within 2 weeks of the index stroke.

#### Primary Outcome

The primary outcome was a reduction in total body weight. The United States Preventive Services Task Force guidelines indicate that a weight reduction of 5% to 10% yields measurable improvement in risk factors for cerebrovascular disease [22]. We measured overall weight reduction in both the smartphone and food journal groups.

We planned a subgroup analysis of weight loss in depression screen–positive and depression screen–negative participants a priori. Poststroke depression is one of the most frequent neuropsychiatric consequences of stroke, affecting approximately 30% of stroke survivors [23]. The presence of poststroke depression has been linked to reduced participation in structured rehabilitation programs [24,25]. Swipe out Stroke was a type of structured dietary rehabilitation intervention; therefore, we analyzed the combined group (smartphone and food journal).

#### Secondary Outcomes

Secondary outcomes included compliance with the weight loss intervention, improvement in depression, and, if abnormal, normalization of systolic blood pressure, serum low-density lipoprotein value, proportion of total hemoglobin, and proportion of serum coagulation factor VIII.

### Data Management

Paper case report forms (screening, feasibility, and tracking forms) were collected and stored in the Institute for Stroke and Cerebrovascular Disease Research Coordinator office at the McGovern Medical School at University of Texas Health Science Center at Houston. We used REDCap version 6.10 (REDCap Consortium) for online form design, data entry, data verification, and data management. All forms were precoded to minimize errors. During data collection, forms were screened upon receipt for completeness.

### Statistical Analysis

We reported descriptive statistics (frequency and percentage for categorical variables, and mean and standard deviation or median and IQR for continuous variables). We used 2-sample *t* test or Wilcoxon rank sum test to compare continuous variables, and Fisher exact test to compare categorical variables.

We obtained demographics and cerebrovascular risk factors by self-report. Cognitive testing was completed at baseline. Depression screening was completed at baseline and days 30, 90, and 180 and compared between the smartphone and food journal groups using Wilcoxon rank sum test. We measured the primary outcome as weight change in pounds; we also compared weight change by the presence of depression (defined as self-reported depression or PHQ-9 score ≥5). We performed all analyses using SAS version 9.4 (SAS Institute); we considered *P*<.05 to be significant.

## Results

### Study Period

Participants were enrolled in Swipe out Stroke between March 2015 and May 2016. The final follow-up visit occurred in December 2016. There were no significant changes in computer hardware or internet delivery resources during the study period. The duration of each in-person study visit was 30 minutes. For the smartphone group, engagement by a member of the study team took approximately 3 minutes per participant during the initiation phase, 2 minutes per participant during the application phase, and 1 minute per participant during the maintenance phase.

### Feasibility and Treatment Fidelity

**Figure 2:**
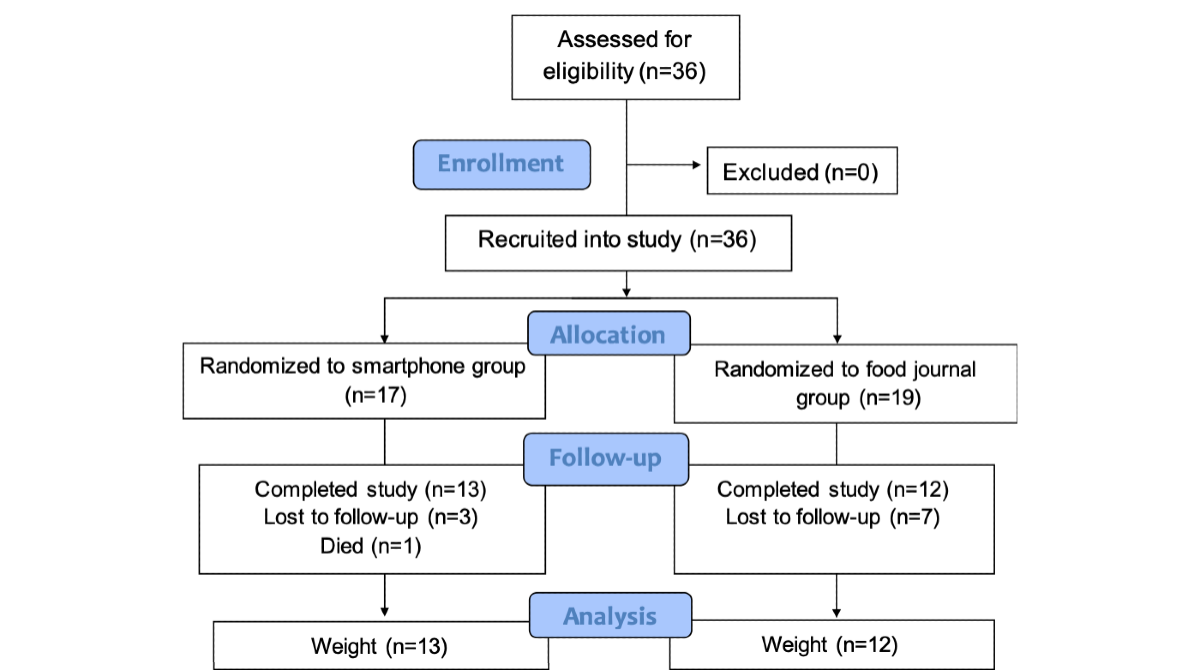
Swipe out Stroke Consolidated Standards of Reporting Trials (CONSORT) flow diagram.

The CONSORT flow diagram in Figure 2 details Swipe out Stroke participant retention. A total of 25 (69%) stroke survivors completed Swipe out Stroke (13/17 in the smartphone group, 12/19 in the food journal group); 1 participant died in the smartphone group. The overall retention rate was 78% (28/36) at day 30, 72% (26/36) at day 90, and 69% (25/36) at day 180.

Intervention adherence was significantly higher at day 90 in the smartphone group than in the food journal group (10/17, 59% vs 4/19, 21%; *P*=.04). In the smartphone group, adherence to 1 self-monitored diet entry per day was 59% (10/17) at day 30 and 35% (6/17) at day 180. Adherence to 1 self-monitored diet entry per day in the food journal group was 42% (8/19) at day 30 and 11% (2/19) at day 180.

### Demographics and Self-Reported Cerebrovascular Risk Factors

Table 1 describes participant demographics and self-reported cerebrovascular risk factors by treatment group. Equal allocation was achieved between the smartphone and food journal groups. The mean participant age was 54 years, and mean education level was completion of 12th grade, some College, or General Education Development. Of the 36 participants, 20 (56%) were male and 23 (64%) were African American. The majority of participants had a source of income, either through retirement or disability (14/36) or employment (self or significant other, 20/36). The participants’ overall median PHQ-9 scores (5.5) signified mild to moderate depression; the median MoCA score (23.5, IQR 21-26) indicated mild cognitive impairment.

**Table 1:** Demographics and self-reported cerebrovascular risk factors.

Characteristics		Total (n=36)		Smartphone (n=17)		Food journal (n=19)		*P* value	
Age (years), mean (SD)		54.1 (9.4)		54.4 (10.9)		53.8 (8.2)		.87^a^	
**Race, n (%)**									
	Black		23 (64)		9 (53)		14 (74)		.30^b^
	Hispanic		13 (36)		8 (47)		5 (26)		
Male sex, n (%)		20 (56)		9 (53)		11 (58)		.77^c^	
**Marital status, n (%)**									
	Married or domestic partner		21 (58)		10 (59)		11 (58)		.96^c^
	Not married		15 (42)		7 (41)		8 (42)		
**Employment status, n (%)**									
	Employed		20 (56)		10 (59)		10 (53)		.87^b^
	Not employed		2 (6)		1 (6)		1 (5)		
	Retired or receiving disability		14 (39)		6 (35)		8 (42)		
**Education, n (%)**									
	Master’s degree		5 (14)		1 (6)		1 (5)		.26^b^
	Bachelor’s degree		3 (8)		0 (0)		3 (16)		
	Associate’s degree or other		21 (58)		4 (24)		1 (5)		
	High school, some college, or GED^d^		5 (14)		9 (53)		12 (63)		
	Less than high school		2 (6)		3 (18)		2 (11)		
**Modified Rankin Scale score, n (%)**									
	0		4 (11)		2 (12)		2 (11)		.92^b^
	1		19 (53)		9 (53)		10 (53)		
	2		8 (22)		3 (18)		5 (26)		
	3		5 (14)		3 (18)		2 (11)		
Patient Health Questionnaire-9, median (IQR)		5.5 (3.0-9.5)		6 (2-10)		5 (3-9)		.74^e^	
**Patient Health Questionnaire-9, n (%)**								.90^b^	
	0-4		17 (48)		8 (47)		9 (47)		
	5-14		14 (39)		6 (35)		8 (42)		
	≥15		5 (14)		3 (18)		2 (11)		
Self-reported depression or psychiatric disease, n (%)		12 (33)		5 (29)		7 (37)		.73^b^	
Montreal Cognitive Assessment, median (IQR)		23.5 (21-26)		23 (19-25)		25 (21-27)		.15^e^	
**Medical history, n (%)**									
	Cardiac disease		11 (31)		4 (24)		7 (37)		.48^b^
	Hypertension		35 (97)		17 (100)		18 (95)		>.99^b^
	Hyperlipidemia		21 (60)		10 (63)		11 (58)		.78^c^
	Family history of obesity		33 (94)		16 (94)		17 (94)		>.99^b^
	Family history of stroke		24 (69)		12 (71)		12 (67)		>.99^b^
	Personal history of stroke		7 (20)		3 (19)		4 (21)		>.99^b^

^a^*P* values obtained by 2-sample *t* test.

^b^*P* values obtained by Fisher exact test.

^c^*P* values obtained by chi-square test.

^d^GED: General Education Development.

^e^*P* values obtained by Wilcoxon rank sum test.

### Objective Cerebrovascular Risk Factors

Table 2 describes objective cerebrovascular risk factors. The mean baseline body mass index was 35.7 (SD 5.7) kg/m^2^, systolic blood pressure was 127.7 (SD 17.0) mm Hg, and serum total cholesterol, high-density lipoprotein, low-density lipoprotein, and triglycerides were in the reference ranges. Median proportion of total hemoglobin (0.061, IQR 0.056-0.07) and proportion of factor VIII (2.29, IQR 1.63-3.09) were both elevated.

**Table 2:** Objective cerebrovascular risk factors.

Risk factors	Total (n=36)	Smartphone (n=17)	Food journal (n=19)	*P* value
Body mass index (kg/m^2^), mean (SD)	35.7 (5.7)	35.1 (5.3)	36.2 (6.2)	.57^a^
Weight (lb), mean (SD)	228.3 (47.1)	219.9 (49.6)	235.8 (44.7)	.32^a^
Systolic blood pressure (mmHg), mean (SD)	127.7 (17.0)	125.1 (9.8)	130.1 (14.1)	.38^a^
Total cholesterol (mmol/L), median (IQR)	9.27 (7.77-10.38)	8.94 (7.21-10.16)	9.43 (7.88-10.43)	.51^b^
Low-density lipoprotein (mmol/L), median (IQR)	4.88 (4.05-6.47)	4.94 (3.94-6.16)	4.77 (4.16-6.55)	.81^b^
High-density lipoprotein (mmol/L), median (IQR)	2.44 (2.0-2.66)	2.44 (2.05-2.66)	2.44 (1.94-2.55)	.51^b^
Triglycerides (mmol/L), median (IQR)	7.83 (5.55-9.63)	7.05 (4.5-9.55)	7.88 (5.83-11.77)	.40^b^
Proportion of total hemoglobin, median (IQR)	0.061 (0.056-0.07)	0.062 (0.056-0.096)	0.06 (0.056-0.065)	.39^b^
Proportion of factor VIII, median (IQR)	2.29 (1.63-3.09)	2.64 (1.68-3.70)	2.15 (1.44-3.04)	.26^b^

^a^*P* values by 2-sample *t* test.

^b^*P* values by Wilcoxon rank sum test.

### Primary Outcome

**Figure 3:**
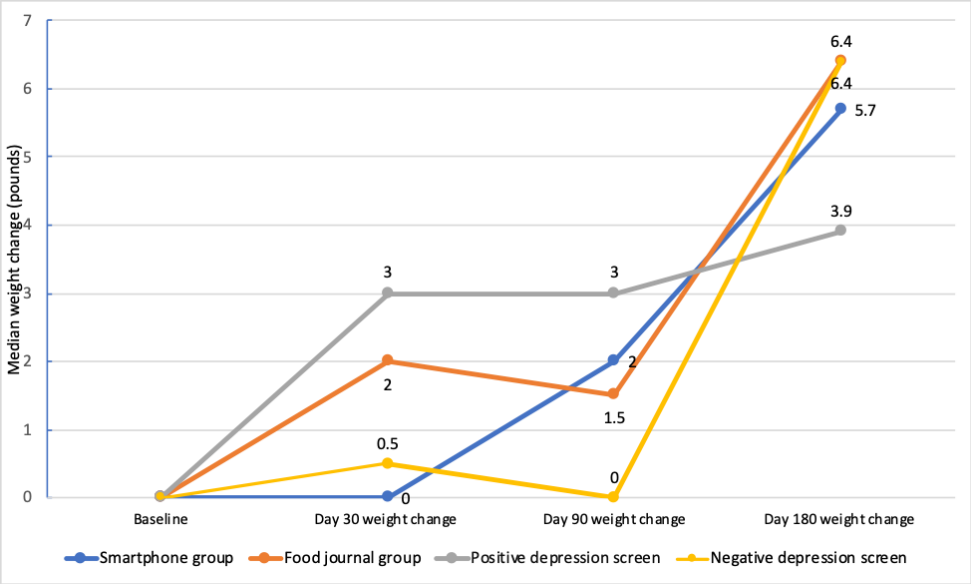
Median weight change by intervention group and depression status.

Figure 3 summarizes descriptive findings on weight outcomes at each study collection time point. There were no statistically significant differences in weight loss between the smartphone and food journal groups. At day 180, the smartphone group had a median weight loss of 5.7 pounds (IQR –2.4 to 8.0), and the food journal group had a median weight loss of 6.4 pounds (IQR –2.2 to 12.5; *P*=.77). There was a 2.1% median weight change in the smartphone group, compared with a 2.9% median weight change in the food journal group (*P*=.63). In the combined smartphone and food journal analysis at day 180, the depression screen–positive group lost 3.9 pounds (IQR –10.1 to 14), and the depression screen–negative group lost 6.4 pounds (IQR –2.4 to 15; *P*=.49).

### Secondary Outcomes

There was no difference in the proportion of total hemoglobin above 0.06 and proportion of factor VIII above 2 in the smartphone and food journal groups at 180 days (*P*=.68).

**Figure 4:**
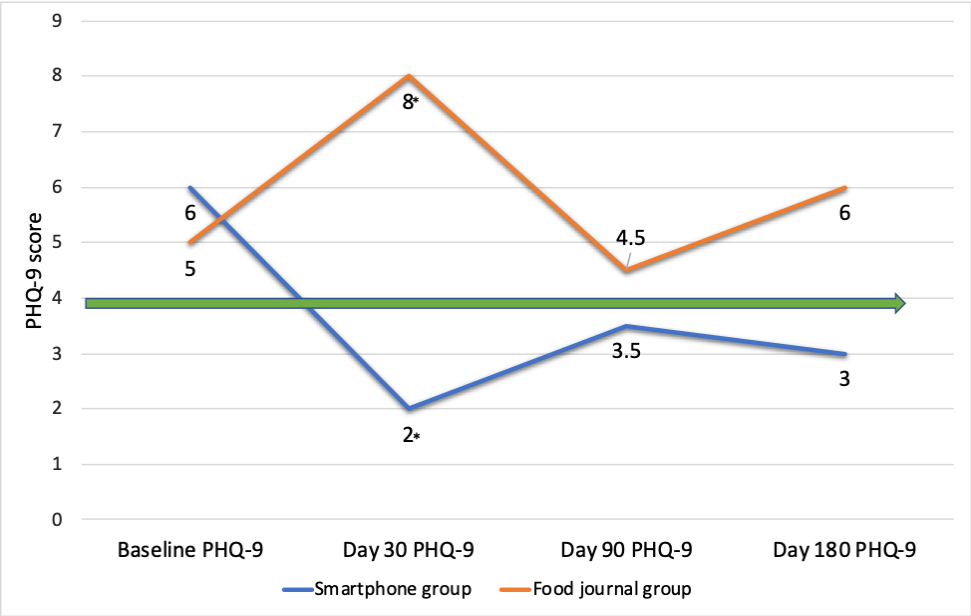
Depression by median Patient Health Questionnaire-9 (PHQ-9) score in the smartphone and food journal groups. Green arrow Indicates zero to minimal depression cutoff (PHQ-9 ≤4). *Statistically significant difference (*P*=.03).

Depression was significantly lower at 30 days in the smartphone group than in the food journal group (PHQ-9 score 2 vs 8; *P*=.03) (Figure 4). Clinically relevant depression rates remained in the zero to minimal range for the smartphone group compared with the mild to moderate range in the food journal group at day 90 (PHQ-9 score 3.5 vs 4.5; *P*=.39) and day 180 (PHQ-9 score 3 vs 6; *P*=.12) (Figure 4). The IQR for PHQ-9 scores remained lower in the smartphone group than in the food journal group at day 30 (smartphone group IQR 1-4 vs food journal group IQR 4-10.5), day 90 (IQR 1-8 vs IQR 3-10), and day 180 (IQR 0-6 vs IQR 1-9).

## Discussion

### Principal Findings

The use of our smartphone-based intervention did not lead to a significant difference in weight change compared with the use of a food journal–based intervention. To our knowledge, this study is the first to implement a self-monitored smartphone-based intervention, with caregiver support, in minority patients with poststroke cognitive impairments. The time interval immediately following an acute stroke presents the opportunity to successfully change dietary patterns in patients and their caregivers, who influence the availability of healthy food choices. The overall retention rates establish an early precedent in this population at increased risk for recurrent stroke. A recent meta-analysis by Lui et al yielded no original clinical trials of the role of mHealth in recurrent stroke prevention [26].

The findings of increased patient engagement and adherence to self-monitoring in the smartphone group are consistent with studies in non–stroke populations [27-31]. Augmenting electronic health tools with non-Web–based components, such as culturally competent dietary counseling, measuring cups, and AHA cookbooks and reading materials, was well received by both the smartphone and food journal groups. Exit interviews reflected appreciation of a template that can be followed to establish healthy eating behaviors. The provision of this heightened supportive care, plus the Hawthorne effect, could have resulted in no significant difference in weight loss between the food journal and smartphone groups.

We found significantly decreased depression rates in the smartphone group at 30 days, which remained in the zero to minimal range; the food journal group had PHQ-9 scores indicative of mild to moderate depression throughout the study. Although both groups had built-in caregiver support, the smartphone group received reminder messages and positive reinforcement on a daily, then weekly, basis, providing support for the weight loss intervention. The use of the Lose It! mobile app as a tool for mitigation of poststroke depression is an interesting, cost-free adjunct in an underresourced minority population. Qu et al found that the top-rated mobile apps for depression were available at a direct cost for more advanced features (up to US $29.99 per month) or an indirect cost in terms of advertisements, which raised privacy concerns [32]. During the first 6 months after a stroke, depression appears to be reactive and is correlated with increased severity of impairment in activities of daily living at 1 year [33]. Kim et al described an improvement in depression at 12 weeks using a smartphone-based mHealth system in a Korean cohort [34]. The effect of direct patient engagement via existing mobile apps, on both poststroke depression and weight loss, is an exciting direction of future studies.

### Limitations

There are several limitations to this study. First, the primary focus was feasibility and early treatment effect; there was not sufficient power to detect group differences. Second, study participants used their personally owned smartphones; during exit interviews, several participants noted financial limitations. The adherence to self-monitoring might have been different if smartphones with data plans had been provided. Third, study participation might have been increased within the smartphone group due to lower rates of depression. We did not examine interactions of cognitive impairment or depression with the treatment effect of each intervention, due to the small sample size in this pilot study. Fourth, our study evaluated short-term outcomes; assessment of the long-term effects of a smartphone-based intervention on weight loss and poststroke depression is a goal for future studies.

### Conclusions

Swipe out Stroke provided useful data on designing subsequent weight management trials for obese minority stroke survivors. Both the smartphone and the food journal interventions resulted in weight loss. Data from Swipe out Stroke suggest that poststroke depression improves with smartphone-based engagement. Future minority research studies that include treatment of poststroke depression and cognitive rehabilitation may positively influence intervention efficacy.

## Appendix

Multimedia Appendix 1 CONSORT-eHEALTH checklist (V 1.6.1).

## Abbreviations

AHA: American Heart Association
CONSORT-EHEALTH: Consolidated Standards of Reporting Trials of Electronic and Mobile Health Applications and Online Telehealth
mHealth: mobile health
MoCA: Montreal Cognitive Assessment
PHQ-9: Patient Health Questionnaire-9
