# Supplementary material for: Use of a Smartphone-Based Mobile App for Weight Management in Obese Minority Stroke Survivors: Pilot Randomized Controlled Trial With Open Blinded End Point
Source: JMIR Mhealth Uhealth. 2020 Apr 22;8(4):e17816. doi: 10.2196/17816 (PMC7203620; doi:10.2196/17816)
Supplement: Multimedia Appendix 1 [file mhealth_v8i4e17816_app1.pdf]

CONSORT-EHEALTH (V 1.6.1) - Submission/Publication Form

The CONSORT-EHEALTH checklist is intended for authors of randomized trials evaluating web-based and Internet-based applications/interventions, including mobile interventions, electronic games (incl multiplayer games), social media, certain telehealth applications, and other interactive and/or networked electronic applications. Some of the items (e.g. all subitems under item 5 - description of the intervention) may also be applicable for other study designs.

The goal of the CONSORT EHEALTH checklist and guideline is to be
a) a guide for reporting for authors of RCTs,
b) to form a basis for appraisal of an ehealth trial (in terms of validity)

CONSORT-EHEALTH items/subitems are MANDATORY reporting items for studies published in the Journal of Medical Internet Research and other journals / scientific societies endorsing the checklist.

Items numbered 1., 2., 3., 4a., 4b etc are original CONSORT or CONSORT-NPT (non-pharmacologic treatment) items.
Items with Roman numerals (i., ii, iii, iv etc.) are CONSORT-EHEALTH extensions/clarifications.

As the CONSORT-EHEALTH checklist is still considered in a formative stage, we would ask that you also RATE ON A SCALE OF 1-5 how important/useful you feel each item is FOR THE PURPOSE OF THE CHECKLIST and reporting guideline (optional).

Mandatory reporting items are marked with a red *.
In the textboxes, either copy & paste the relevant sections from your manuscript into this form - please include any quotes from your manuscript in QUOTATION MARKS,
or answer directly by providing additional information not in the manuscript, or elaborating on why the item was not relevant for this study.

YOUR ANSWERS WILL BE PUBLISHED AS A SUPPLEMENTARY FILE TO YOUR PUBLICATION IN JMIR AND ARE CONSIDERED PART OF YOUR PUBLICATION (IF ACCEPTED).
Please fill in these questions diligently. Information will not be copyedited, so please use proper spelling and grammar, use correct capitalization, and avoid abbreviations.

DO NOT FORGET TO SAVE AS PDF _AND_ CLICK THE SUBMIT BUTTON SO YOUR ANSWERS ARE IN OUR DATABASE !!!

Citation Suggestion (if you append the pdf as Appendix we suggest to cite this paper in the caption):
Eysenbach G, CONSORT-EHEALTH Group
CONSORT-EHEALTH: Improving and Standardizing Evaluation Reports of Web-based and

Mobile Health Interventions
J Med Internet Res 2011;13(4):e126
URL: http://www.jmir.org/2011/4/e126/
doi: 10.2196/jmir.1923
PMID: 22209829

* Required

Your name *

First Last

Nneka Ifejika

Primary Affiliation (short), City, Country *

University of Toronto, Toronto, Canada

UT Southwestern Medical Center, Houston, US

Your e-mail address *

abc@gmail.com

nneka.ifejika@utsouthwestern.edu

Title of your manuscript *

Provide the (draft) title of your manuscript.

Using a Smartphone-based Mobile
Application for Weight Management in Obese Minority Stroke
Survivors: Pilot Randomized Controlled Trial with Open Blinded Endpoint

Name of your App/Software/Intervention *

If there is a short and a long/alternate name, write the short name first and add the long name in brackets.

Lose it!

Evaluated Version (if any)

e.g. "V1", "Release 2017-03-01", "Version 2.0.27913"

Version 5.2.1, Released 03/07/2015

Language(s) *

What language is the intervention/app in? If multiple languages are available, separate by comma (e.g. "English, French")

English

URL of your Intervention Website or App

e.g. a direct link to the mobile app on app in appstore (itunes, Google Play), or URL of the website. If the intervention is a DVD or hardware, you can also link to an Amazon page.

https://www.loseit.com/

URL of an image/screenshot (optional)

Your answer

Accessibility *

Can an enduser access the intervention presently?

- (selected) access is free and open
- access only for special usergroups, not open
- access is open to everyone, but requires payment/subscription/in-app purchases
- app/intervention no longer accessible
- Other:

Primary Medical Indication/Disease/Condition *

e.g. "Stress", "Diabetes", or define the target group in brackets after the condition, e.g. "Autism (Parents of children with)", "Alzheimers (Informal Caregivers of)"

Stroke (Obesity in Minorities with)

Primary Outcomes measured in trial *

comma-separated list of primary outcomes reported in the trial

Weight Loss

Secondary/other outcomes

Are there any other outcomes the intervention is expected to affect?

Your answer

Recommended "Dose" *

What do the instructions for users say on how often the app should be used?

- (selected) Approximately Daily
- Approximately Weekly
- Approximately Monthly
- Approximately Yearly
- "as needed"
- Other:

Approx. Percentage of Users (starters) still using the app as recommended after 3 months *

- unknown / not evaluated
- 0-10%
- 11-20%
- 21-30%
- 31-40%
- 41-50%
- (selected) 51-60%
- 61-70%
- 71%-80%
- 81-90%
- 91-100%
- Other:

Overall, was the app/intervention effective? *

- ○ yes: all primary outcomes were significantly better in intervention group vs control
- ○ partly: SOME primary outcomes were significantly better in intervention group vs control
- ◉ no statistically significant difference between control and intervention
- ○ potentially harmful: control was significantly better than intervention in one or more outcomes
- ○ inconclusive: more research is needed
- ○ Other:

Article Preparation Status/Stage *

At which stage in your article preparation are you currently (at the time you fill in this form)

- ○ not submitted yet - in early draft status
- ○ not submitted yet - in late draft status, just before submission
- ◉ submitted to a journal but not reviewed yet
- ○ submitted to a journal and after receiving initial reviewer comments
- ○ submitted to a journal and accepted, but not published yet
- ○ published
- ○ Other:

Journal *

If you already know where you will submit this paper (or if it is already submitted), please provide the journal name (if it is not JMIR, provide the journal name under "other")

- ( ) not submitted yet / unclear where I will submit this
- ( ) Journal of Medical Internet Research (JMIR)
- (•) JMIR mHealth and UHealth
- ( ) JMIR Serious Games
- ( ) JMIR Mental Health
- ( ) JMIR Public Health
- ( ) JMIR Formative Research
- ( ) Other JMIR sister journal
- ( ) Other:

Is this a full powered effectiveness trial or a pilot/feasibility trial? *

- (•) Pilot/feasibility
- ( ) Fully powered

Manuscript tracking number *

If this is a JMIR submission, please provide the manuscript tracking number under "other" (The ms tracking number can be found in the submission acknowledgement email, or when you login as author in JMIR. If the paper is already published in JMIR, then the ms tracking number is the four-digit number at the end of the DOI, to be found at the bottom of each published article in JMIR)

- (•) no ms number (yet) / not (yet) submitted to / published in JMIR
- ( ) Other:

TITLE AND ABSTRACT

1a) TITLE: Identification as a randomized trial in the title

1a) Does your paper address CONSORT item 1a? *

I.e does the title contain the phrase "Randomized Controlled Trial"? (if not, explain the reason under "other")

- (x) yes
- ( ) Other:

1a-i) Identify the mode of delivery in the title

Identify the mode of delivery. Preferably use “web-based” and/or “mobile” and/or “electronic game” in the title. Avoid ambiguous terms like “online”, “virtual”, “interactive”. Use “Internet-based” only if Intervention includes non-web-based Internet components (e.g. email), use “computer-based” or “electronic” only if offline products are used. Use “virtual” only in the context of “virtual reality” (3-D worlds). Use “online” only in the context of “online support groups”. Complement or substitute product names with broader terms for the class of products (such as “mobile” or “smart phone” instead of “iphone”), especially if the application runs on different platforms.

	1	2	3	4	5	
subitem not at all important	( )	( )	( )	( )	(x)	essential

Does your paper address subitem 1a-i? *

Copy and paste relevant sections from manuscript title (include quotes in quotation marks "like this" to indicate direct quotes from your manuscript), or elaborate on this item by providing additional information not in the ms, or briefly explain why the item is not applicable/relevant for your study

Using a Smartphone-based Mobile
Application for Weight Management in Obese Minority Stroke
Survivors: Pilot Randomized Controlled Trial with Open Blinded Endpoint

1a-ii) Non-web-based components or important co-interventions in title

Mention non-web-based components or important co-interventions in title, if any (e.g., “with telephone support”).

	1	2	3	4	5	
subitem not at all important	◉	○	○	○	○	essential

Does your paper address subitem 1a-ii?

Copy and paste relevant sections from manuscript title (include quotes in quotation marks "like this" to indicate direct quotes from your manuscript), or elaborate on this item by providing additional information not in the ms, or briefly explain why the item is not applicable/relevant for your study

There were no co-interventions.

1a-iii) Primary condition or target group in the title

Mention primary condition or target group in the title, if any (e.g., “for children with Type I Diabetes”) Example: A Web-based and Mobile Intervention with Telephone Support for Children with Type I Diabetes: Randomized Controlled Trial

	1	2	3	4	5	
subitem not at all important	○	○	○	○	◉	essential

Does your paper address subitem 1a-iii? *

Copy and paste relevant sections from manuscript title (include quotes in quotation marks "like this" to indicate direct quotes from your manuscript), or elaborate on this item by providing additional information not in the ms, or briefly explain why the item is not applicable/relevant for your study

Using a Smartphone-based Mobile
Application for Weight Management in Obese Minority Stroke
Survivors: Pilot Randomized Controlled Trial with Open Blinded Endpoint

1b) ABSTRACT: Structured summary of trial design, methods, results, and conclusions

NPT extension: Description of experimental treatment, comparator, care providers, centers, and blinding status.

1b-i) Key features/functionalities/components of the intervention and comparator in the METHODS section of the ABSTRACT

Mention key features/functionalities/components of the intervention and comparator in the abstract. If possible, also mention theories and principles used for designing the site. Keep in mind the needs of systematic reviewers and indexers by including important synonyms. (Note: Only report in the abstract what the main paper is reporting. If this information is missing from the main body of text, consider adding it)

	1	2	3	4	5	
subitem not at all important	○	○	○	○	◉	essential

Does your paper address subitem 1b-i? *

Copy and paste relevant sections from the manuscript abstract (include quotes in quotation marks "like this" to indicate direct quotes from your manuscript), or elaborate on this item by providing additional information not in the ms, or briefly explain why the item is not applicable/relevant for your study

"Swipe out Stroke is a pilot Prospective Randomized Controlled Trial with Open Blinded Endpoint (PROBE) study.

After written informed consent was obtained, both patients and their caregivers were screened for vascular risk factors using cluster enrollment at a Joint Commission Comprehensive Stroke Center in Houston Texas.

Adaptive randomization was used for assignment to one of two groups – 1) behavior intervention with SmartPhone based self-monitoring, 2) behavior intervention with food journal self-monitoring. Caregivers joined the group of the study participant.
The SmartPhone group received daily reminder messages during the first 30 days, reminder messages on missed days plus weekly summaries between 31 and 90 days, and weekly summaries only between 91 and 180 days."

1b-ii) Level of human involvement in the METHODS section of the ABSTRACT

Clarify the level of human involvement in the abstract, e.g., use phrases like "fully automated" vs. "therapist/nurse/care provider/physician-assisted" (mention number and expertise of providers involved, if any). (Note: Only report in the abstract what the main paper is reporting. If this information is missing from the main body of text, consider adding it)

	1	2	3	4	5	
subitem not at all important	○	○	○	○	◉	essential

Does your paper address subitem 1b-ii?

Copy and paste relevant sections from the manuscript abstract (include quotes in quotation marks "like this" to indicate direct quotes from your manuscript), or elaborate on this item by providing additional information not in the ms, or briefly explain why the item is not applicable/relevant for your study

"Both the SmartPhone and food journal groups received four face-to-face visits (baseline, 30 days, 90 days, 180 days), culturally competent counseling by a minority research coordinator, cookbooks, measuring cups and educational materials."

1b-iii) Open vs. closed, web-based (self-assessment) vs. face-to-face assessments in the METHODS section of the ABSTRACT

Mention how participants were recruited (online vs. offline), e.g., from an open access website or from a clinic or a closed online user group (closed usergroup trial), and clarify if this was a purely web-based trial, or there were face-to-face components (as part of the intervention or for assessment). Clearly say if outcomes were self-assessed through questionnaires (as common in web-based trials). Note: In traditional offline trials, an open trial (open-label trial) is a type of clinical trial in which both the researchers and participants know which treatment is being administered. To avoid confusion, use "blinded" or "unblinded" to indicated the level of blinding instead of "open", as "open" in web-based trials usually refers to "open access" (i.e. participants can self-enrol). (Note: Only report in the abstract what the main paper is reporting. If this information is missing from the main body of text, consider adding it)

	1	2	3	4	5	
subitem not at all important	○	○	○	◉	○	essential

Does your paper address subitem 1b-iii?

Copy and paste relevant sections from the manuscript abstract (include quotes in quotation marks "like this" to indicate direct quotes from your manuscript), or elaborate on this item by providing additional information not in the ms, or briefly explain why the item is not applicable/relevant for your study

"After written informed consent was obtained, both patients and their caregivers were screened for vascular risk factors using cluster enrollment at a Joint Commission Comprehensive Stroke Center in Houston Texas."

1b-iv) RESULTS section in abstract must contain use data

Report number of participants enrolled/assessed in each group, the use/uptake of the intervention (e.g., attrition/adherence metrics, use over time, number of logins etc.), in addition to primary/secondary outcomes. (Note: Only report in the abstract what the main paper is reporting. If this information is missing from the main body of text, consider adding it)

	1	2	3	4	5	
subitem not at all important	○	○	○	○	◉	essential

Does your paper address subitem 1b-iv?

Copy and paste relevant sections from the manuscript abstract (include quotes in quotation marks "like this" to indicate direct quotes from your manuscript), or elaborate on this item by providing additional information not in the ms, or briefly explain why the item is not applicable/relevant for your study

"Thirty-six stroke patients (64% African-American, 36% Hispanic) were enrolled, 17 in the SmartPhone group, 19 in the food journal group. Mean age 54 (SD 9) years, BMI 35.7 (SD 5.7) education, employment status and family history of stroke or obesity did not differ between the groups.
Baseline rates of depression [median Patient Health Questionnaire-9 (PHQ-9) 5.5; IQR 3.0 – 9.5), cognitive impairment [median Montreal Cognitive Assessment (MOCA) 23.5; IQR 21 – 26] and inability to ambulate (13% with mRS of 3) were similar.

Sixty-nine percent of stroke survivors completed Swipe out Stroke (n=25, 13/17 in the SmartPhone group, 12/19 in the food journal group); one participant died in the SmartPhone group.
The median weight change at 180 days was 5.7 pounds (IQR -2.4 to 8.0) in the SmartPhone group versus 6.4 pounds in the food journal group (IQR -2.2 to 12.5; P=0.77).

Depression was significantly lower at 30 days in the SmartPhone group compared to the food journal group (PHQ-9 of 2 vs 8; P=0.03). Clinically relevant depression rates remained in the “zero to minimal” range for the SmartPhone group compared to “mild to moderate” range in the food journal group at Day 90 (PHQ-9 of 3.5 vs 4.5; P=0.39) and Day 180 (PHQ-9 of 3 vs 6; P=0.12)."

1b-v) CONCLUSIONS/DISCUSSION in abstract for negative trials

Conclusions/Discussions in abstract for negative trials: Discuss the primary outcome - if the trial is negative (primary outcome not changed), and the intervention was not used, discuss whether negative results are attributable to lack of uptake and discuss reasons. (Note: Only report in the abstract what the main paper is reporting. If this information is missing from the main body of text, consider adding it)

	1	2	3	4	5	
subitem not at all important	○	○	○	◉	○	essential

Does your paper address subitem 1b-v?

Copy and paste relevant sections from the manuscript abstract (include quotes in quotation marks "like this" to indicate direct quotes from your manuscript), or elaborate on this item by providing additional information not in the ms, or briefly explain why the item is not applicable/relevant for your study

"In our patient population of obese minority stroke survivors with cognitive impairments, the use of a SmartPhone did not lead to a significant reduction in weight loss compared to a food journal. The presence of baseline depression (53%) was a confounding variable, which improved with SmartPhone engagement. Future studies that include treatment of post-stroke depression may positively influence intervention efficacy. "

INTRODUCTION

2a) In INTRODUCTION: Scientific background and explanation of rationale

2a-i) Problem and the type of system/solution

Describe the problem and the type of system/solution that is object of the study: intended as stand-alone intervention vs. incorporated in broader health care program? Intended for a particular patient population? Goals of the intervention, e.g., being more cost-effective to other interventions, replace or complement other solutions? (Note: Details about the intervention are provided in “Methods” under 5)

	1	2	3	4	5	
subitem not at all important	○	○	○	○	◉	essential

Does your paper address subitem 2a-i? *

Copy and paste relevant sections from the manuscript (include quotes in quotation marks "like this" to indicate direct quotes from your manuscript), or elaborate on this item by providing additional information not in the ms, or briefly explain why the item is not applicable/relevant for your study

"There are limited data on the use of mHealth interventions for weight loss in stroke patients. Stroke affects the cognitive domains of attention, memory, language and orientation [ ]; approximately 30% of stroke patients develop dementia within 1 year of stroke onset [ ]. Weight loss interventions that are accessible and easy to use for stroke patients with residual cognitive impairments may help mitigate the burden of recurrent cerebrovascular disease.
Swipe out Stroke (SOS) tested the feasibility and preliminary treatment effects of using a SmartPhone-based dietary intervention in obese minority patients, many of whom with post-stroke cognitive impairments. Svetkey et al. [ ] showed that personal contact with a healthcare professional is the most effective means to sustain weight loss. SOS facilitated personal contact through the use of a mobile application, providing positive reinforcement of dietary modifications, and giving support when participants experienced difficulty with program maintenance."

2a-ii) Scientific background, rationale: What is known about the (type of) system

Scientific background, rationale: What is known about the (type of) system that is the object of the study (be sure to discuss the use of similar systems for other conditions/diagnoses, if appropiate), motivation for the study, i.e. what are the reasons for and what is the context for this specific study, from which stakeholder viewpoint is the study performed, potential impact of findings [2]. Briefly justify the choice of the comparator.

	1	2	3	4	5	
subitem not at all important	○	○	○	◉	○	essential

Does your paper address subitem 2a-ii? *

Copy and paste relevant sections from the manuscript (include quotes in quotation marks "like this" to indicate direct quotes from your manuscript), or elaborate on this item by providing additional information not in the ms, or briefly explain why the item is not applicable/relevant for your study

"Decades of research have shown a disproportionate incidence of obesity among African-American and Hispanic populations [, ,]. Highly correlated with obesity, minority populations have a median age of first stroke 10–13 years earlier than non-Hispanic Caucasians [,].
Adherence to evidence-based therapies for the management of obesity in minority populations at risk of stroke remains insufficient. Previous studies have demonstrated success in targeting obese minority patients through restrictive health promotion interventions [ ] and stroke navigators [ ] both of which require close follow-up by a medical professional. Unfortunately, inadequate finances and lack of health insurance are barriers to care in minority patients[ ] therefore, study protocols that require frequent medical follow-up are not easily generalizable.

Viable options to bypass this barrier are studies that utilize self-monitoring of caloric intake. Minority ethnicity has a positive influence on self-monitoring behaviors [ ]. Hollis et al. conducted a nonrandomized behavioral intervention, evaluating the effects of caloric restriction, moderate physical activity and the DASH (Dietary Approaches to Stop Hypertension) diet on weight loss. The number of food records completed had a stronger association with weight loss among African-Americans when compared to non–African-Americans, regardless of sex [ ] In addition, racial differences in weight loss between African-Americans and non–African-Americans were less pronounced than in previous studies, a finding that could be attributed to African-American representation among investigators, staff and study participants.

The feasibility of using electronic devices to self-monitor caloric intake has been established in the literature. Yon et al. [ ] conducted a study of nonconcurrent groups, one using a personal digital assistant (PDA), the other using a paper diary. Both methods were comparable, with similar weight loss between the groups. In the SMART trial, Burke et al [9,] tested the use of a PDA with calorie monitoring software; PDA use was more socially acceptable than a paper diary, and participants did not encounter major difficulties in learning how to use the software. Beasley et al. [ ] reported that new users of calorie monitoring software took 8–10 minutes to enter a meal, which is similar to recording a meal in a paper diary. While the PDA has become obsolete, many of its features are incorporated into smartphones, and the associated calorie monitoring software has become readily available."

2b) In INTRODUCTION: Specific objectives or hypotheses

Does your paper address CONSORT subitem 2b? *

Copy and paste relevant sections from the manuscript (include quotes in quotation marks "like this" to indicate direct quotes from your manuscript), or elaborate on this item by providing additional information not in the ms, or briefly explain why the item is not applicable/relevant for your study

"Swipe out Stroke (SOS) tested the feasibility and preliminary treatment effects of using a SmartPhone-based dietary intervention in obese minority patients, many of whom with post-stroke cognitive impairments."

METHODS

3a) Description of trial design (such as parallel, factorial) including allocation ratio

Does your paper address CONSORT subitem 3a? *

Copy and paste relevant sections from the manuscript (include quotes in quotation marks "like this" to indicate direct quotes from your manuscript), or elaborate on this item by providing additional information not in the ms, or briefly explain why the item is not applicable/relevant for your study

"Swipe out Stroke was a Phase I, pilot, prospective, randomized controlled trial with open-blinded endpoint (PROBE), testing the feasibility and preliminary treatment effects of using a SmartPhone based dietary intervention in obese minority patients with a heightened risk of post-stroke cognitive impairments. "

3b) Important changes to methods after trial commencement (such as eligibility criteria), with reasons

Does your paper address CONSORT subitem 3b? *

Copy and paste relevant sections from the manuscript (include quotes in quotation marks "like this" to indicate direct quotes from your manuscript), or elaborate on this item by providing additional information not in the ms, or briefly explain why the item is not applicable/relevant for your study

We did not change the eligibility criteria. The inclusion and exclusion criteria are listed in Table 1 of the manuscript.

3b-i) Bug fixes, Downtimes, Content Changes

Bug fixes, Downtimes, Content Changes: ehealth systems are often dynamic systems. A description of changes to methods therefore also includes important changes made on the intervention or comparator during the trial (e.g., major bug fixes or changes in the functionality or content) (5-iii) and other “unexpected events” that may have influenced study design such as staff changes, system failures/downtimes, etc. [2].

	1	2	3	4	5	
subitem not at all important	◉	○	○	○	○	essential

Does your paper address subitem 3b-i?

Copy and paste relevant sections from the manuscript (include quotes in quotation marks "like this" to indicate direct quotes from your manuscript), or elaborate on this item by providing additional information not in the ms, or briefly explain why the item is not applicable/relevant for your study

There were no major bug fixes to Lose it! or staff changes during the study period.

4a) Eligibility criteria for participants

Does your paper address CONSORT subitem 4a? *

Copy and paste relevant sections from the manuscript (include quotes in quotation marks "like this" to indicate direct quotes from your manuscript), or elaborate on this item by providing additional information not in the ms, or briefly explain why the item is not applicable/relevant for your study

Data from Table 1 - Inclusion and Exclusion Criteria:

"Inclusion Criteria
1. Ischemic or hemorrhagic stroke.
2. Age 18-85 years.
3. African-American or Hispanic.
4. Post-stroke Modified Rankin Scale (mRS) of 0-3.
5. Post-stroke Body Mass Index greater than 30 kg/m2.
6. Taking prescription medication for diabetes mellitus, hypertension or hyperlipidemia after the cerebrovascular event.
7. Willing to follow a healthy eating pattern and NOT use weight loss medications for the duration of the study.
8. Personal ownership or caregiver ownership of a personal computer, smartphone or other smart device (iPhone or Android platform) with Internet access.
9. If patient has alexia, agraphia, acalculia, dementia or blindness – caregiver must be willing to complete the intervention.

Exclusion Criteria
1. Pre-existing disability with mRS > 4.
2. Contraindications to weight loss (planning to become pregnant, history of an eating disorder).
3. Steroid use for suspected vasculitis.
4. Current or recent (past 6 months) participation in a weight loss program or use of weight loss medication."

4a-i) Computer / Internet literacy

Computer / Internet literacy is often an implicit “de facto” eligibility criterion - this should be explicitly clarified.

	1	2	3	4	5	
subitem not at all important	○	○	○	○	◉	essential

Does your paper address subitem 4a-i?

Copy and paste relevant sections from the manuscript (include quotes in quotation marks "like this" to indicate direct quotes from your manuscript), or elaborate on this item by providing additional information not in the ms, or briefly explain why the item is not applicable/relevant for your study

SmartPhone group participants used their existing devices, the app functionality was reviewed during the baseline visit:

"During the baseline visit, a user account for the mobile application (Lose It!) was created using the participant's e-mail address. Each participant and caregiver (if applicable) received a tutorial on how to download and use the Lose It! on his or her personally owned SmartPhone."

4a-ii) Open vs. closed, web-based vs. face-to-face assessments:

Open vs. closed, web-based vs. face-to-face assessments: Mention how participants were recruited (online vs. offline), e.g., from an open access website or from a clinic, and clarify if this was a purely web-based trial, or there were face-to-face components (as part of the intervention or for assessment), i.e., to what degree got the study team to know the participant. In online-only trials, clarify if participants were quasi-anonymous and whether having multiple identities was possible or whether technical or logistical measures (e.g., cookies, email confirmation, phone calls) were used to detect/prevent these.

	1	2	3	4	5	
subitem not at all important	○	○	○	○	◉	essential

Does your paper address subitem 4a-ii? *

Copy and paste relevant sections from the manuscript (include quotes in quotation marks "like this" to indicate direct quotes from your manuscript), or elaborate on this item by providing additional information not in the ms, or briefly explain why the item is not applicable/relevant for your study

"Obese African-American or Hispanic patients age 18 and older, acutely hospitalized for ischemic or hemorrhagic stroke, were screened for the study. The setting was a Joint Commission certified Comprehensive Stroke Center in Houston Texas with a 200 mile Telemedicine radius, reaching both urban and rural areas with a large underinsured population."

4a-iii) Information giving during recruitment

Information given during recruitment. Specify how participants were briefed for recruitment and in the informed consent procedures (e.g., publish the informed consent documentation as appendix, see also item X26), as this information may have an effect on user self-selection, user expectation and may also bias results.

	1	2	3	4	5	
subitem not at all important	○	○	○	○	◉	essential

Does your paper address subitem 4a-iii?

Copy and paste relevant sections from the manuscript (include quotes in quotation marks "like this" to indicate direct quotes from your manuscript), or elaborate on this item by providing additional information not in the ms, or briefly explain why the item is not applicable/relevant for your study

"Participants were screened by the Stroke Social Worker and a member of the SOS research team during the acute hospitalization. After informed consent was obtained, the baseline clinic visit occurred within 2 weeks of hospital discharge."

4b) Settings and locations where the data were collected

Does your paper address CONSORT subitem 4b? *

Copy and paste relevant sections from the manuscript (include quotes in quotation marks "like this" to indicate direct quotes from your manuscript), or elaborate on this item by providing additional information not in the ms, or briefly explain why the item is not applicable/relevant for your study

"The case report forms (screening, feasibility and tracking forms) were collected and stored in the Institute for Cerebrovascular Disease Research Coordinator office at the McGovern Medical School at UTHealth in Houston."

4b-i) Report if outcomes were (self-)assessed through online questionnaires

Clearly report if outcomes were (self-)assessed through online questionnaires (as common in web-based trials) or otherwise.

	1	2	3	4	5	
subitem not at all important	○	○	○	○	◉	essential

Does your paper address subitem 4b-i? *

Copy and paste relevant sections from the manuscript (include quotes in quotation marks "like this" to indicate direct quotes from your manuscript), or elaborate on this item by providing additional information not in the ms, or briefly explain why the item is not applicable/relevant for your study

"RedCAP Version 6.10 (released 11/25/2015) was used for form design, data entry, data verification and data management. All forms were precoded to minimize errors. During data collection, forms were screened upon receipt for completeness. "

4b-ii) Report how institutional affiliations are displayed

Report how institutional affiliations are displayed to potential participants [on ehealth media], as affiliations with prestigious hospitals or universities may affect volunteer rates, use, and reactions with regards to an intervention.(Not a required item – describe only if this may bias results)

	1	2	3	4	5	
subitem not at all important	◉	○	○	○	○	essential

Does your paper address subitem 4b-ii?

Copy and paste relevant sections from the manuscript (include quotes in quotation marks "like this" to indicate direct quotes from your manuscript), or elaborate on this item by providing additional information not in the ms, or briefly explain why the item is not applicable/relevant for your study

"The setting was a Joint Commission certified Comprehensive Stroke Center in Houston Texas with a 200 mile Telemedicine radius, reaching both urban and rural areas with a large underinsured population."

5) The interventions for each group with sufficient details to allow replication, including how and when they were actually administered

5-i) Mention names, credential, affiliations of the developers, sponsors, and owners

Mention names, credential, affiliations of the developers, sponsors, and owners [6] (if authors/evaluators are owners or developer of the software, this needs to be declared in a “Conflict of interest” section or mentioned elsewhere in the manuscript).

	1	2	3	4	5	
subitem not at all important	○	○	○	○	◉	essential

Does your paper address subitem 5-i?

Copy and paste relevant sections from the manuscript (include quotes in quotation marks "like this" to indicate direct quotes from your manuscript), or elaborate on this item by providing additional information not in the ms, or briefly explain why the item is not applicable/relevant for your study

"During the baseline visit, a user account for the mobile application (Lose It!) was created using the participant's e-mail address. Each participant and caregiver (if applicable) received a tutorial on how to download and use the Lose It! on his or her personally owned SmartPhone.
For each participant, a ten percent weight loss goal was implemented, and a value for maximum daily caloric intake was provided under the "budget" column (Figure 1). SmartPhone group participants could search for foods, including restaurants, or scan a barcode, which uploaded the food product directly onto the Lose It! platform.
The number of calories consumed could be increased with physical activity, logged in the "exercise" column. The "net" calories each day equaled the total of food minus exercise. All SOS participants were provided premium features free of charge for six months, including the food journal group, after the study period.

During the Initiation Phase (Baseline to Day 30), a pattern of compliance with Lose It! use was initiated. Participants received daily monitoring of caloric intake, with reminder messages via push notifications through the mobile application. We anticipated establishment of self-monitoring patterns during the Application Phase (Day 31 to 90). To facilitate positive reinforcement, the SmartPhone group received weekly push notification summaries of compliance, and reminder messages on missed days. The Maintenance Phase 3 (Day 91 to 180) consisted of weekly summaries with no reminder messages. "

5-ii) Describe the history/development process

Describe the history/development process of the application and previous formative evaluations (e.g., focus groups, usability testing), as these will have an impact on adoption/use rates and help with interpreting results.

	1	2	3	4	5	
subitem not at all important	◉	○	○	○	○	essential

Does your paper address subitem 5-ii?

Copy and paste relevant sections from the manuscript (include quotes in quotation marks "like this" to indicate direct quotes from your manuscript), or elaborate on this item by providing additional information not in the ms, or briefly explain why the item is not applicable/relevant for your study

The study used an app that is readily available and free to the public for use.

5-iii) Revisions and updating

Revisions and updating. Clearly mention the date and/or version number of the application/intervention (and comparator, if applicable) evaluated, or describe whether the intervention underwent major changes during the evaluation process, or whether the development and/or content was “frozen” during the trial. Describe dynamic components such as news feeds or changing content which may have an impact on the replicability of the intervention (for unexpected events see item 3b).

	1	2	3	4	5	
subitem not at all important	(●)	( )	( )	( )	( )	essential

Does your paper address subitem 5-iii?

Copy and paste relevant sections from the manuscript (include quotes in quotation marks "like this" to indicate direct quotes from your manuscript), or elaborate on this item by providing additional information not in the ms, or briefly explain why the item is not applicable/relevant for your study

Version 5.2.1 of Lose it!, released 3/7/2015 was used. Updates to Lose it! did not affect the functionality of the software, there were no "frozen" periods.

5-iv) Quality assurance methods

Provide information on quality assurance methods to ensure accuracy and quality of information provided [1], if applicable.

	1	2	3	4	5	
subitem not at all important	( )	( )	( )	( )	(●)	essential

Does your paper address subitem 5-iv?

Copy and paste relevant sections from the manuscript (include quotes in quotation marks "like this" to indicate direct quotes from your manuscript), or elaborate on this item by providing additional information not in the ms, or briefly explain why the item is not applicable/relevant for your study

"General Health and Sociodemographic Data
Participants' age, sex, ethnicity, marital status, employment status, educational level, weight and height (to calculate Body Mass Index) and stroke related disability level were collected in a sociodemographic questionnaire. Cognitive impairments were assessed using the Montreal Cognitive Assessment, a score of 26 and higher was considered normal [ ]. Personal health, family history and past medical history information was collected using a general health history form.

Objective Health Measures
Cardiovascular risk factors were collected in both groups, with normative ranges derived from AHA guidelines [,]. Baseline study measurements were collected within two weeks of the index stroke.

The case report forms (screening, feasibility and tracking forms) were collected and stored in the Institute for Cerebrovascular Disease Research Coordinator office at the McGovern Medical School at UTHealth in Houston. RedCAP Version 6.10 (released 11/25/2015) was used for form design, data entry, data verification and data management. All forms were precoded to minimize errors. During data collection, forms were screened upon receipt for completeness."

5-v) Ensure replicability by publishing the source code, and/or providing screenshots/screen-capture video, and/or providing flowcharts of the algorithms used

Ensure replicability by publishing the source code, and/or providing screenshots/screen-capture video, and/or providing flowcharts of the algorithms used. Replicability (i.e., other researchers should in principle be able to replicate the study) is a hallmark of scientific reporting.

	1	2	3	4	5	
subitem not at all important	○	○	○	○	◉	essential

Does your paper address subitem 5-v?

Copy and paste relevant sections from the manuscript (include quotes in quotation marks "like this" to indicate direct quotes from your manuscript), or elaborate on this item by providing additional information not in the ms, or briefly explain why the item is not applicable/relevant for your study

We have described the SmartPhone and Food Journal interventions in detail in the following protocol publication, which is referenced in the intervention section of this manuscript.

The publication is: Ifejika NL, Noser EA, Grotta JC, Savitz SI. Swipe out Stroke: Feasibility and efficacy of using a smart-phone based mobile application to improve compliance with weight loss in obese minority stroke patients and their carers. Int J Stroke. 2016;11:593-603.

5-vi) Digital preservation

Digital preservation: Provide the URL of the application, but as the intervention is likely to change or disappear over the course of the years; also make sure the intervention is archived (Internet Archive, webcitation.org, and/or publishing the source code or screenshots/videos alongside the article). As pages behind login screens cannot be archived, consider creating demo pages which are accessible without login.

	1	2	3	4	5	
subitem not at all important	○	○	○	○	◉	essential

Does your paper address subitem 5-vi?

Copy and paste relevant sections from the manuscript (include quotes in quotation marks "like this" to indicate direct quotes from your manuscript), or elaborate on this item by providing additional information not in the ms, or briefly explain why the item is not applicable/relevant for your study

In the intervention section of the manuscript:
"During the baseline visit, a user account for the mobile application (Lose It! Version 5.2.1, released 03/07/2015, www.loseit.com) was created using the participant's e-mail address."

5-vii) Access

Access: Describe how participants accessed the application, in what setting/context, if they had to pay (or were paid) or not, whether they had to be a member of specific group. If known, describe how participants obtained “access to the platform and Internet” [1]. To ensure access for editors/reviewers/readers, consider to provide a “backdoor” login account or demo mode for reviewers/readers to explore the application (also important for archiving purposes, see vi).

	1	2	3	4	5	
subitem not at all important	○	○	○	○	◉	essential

Does your paper address subitem 5-vii? *

Copy and paste relevant sections from the manuscript (include quotes in quotation marks "like this" to indicate direct quotes from your manuscript), or elaborate on this item by providing additional information not in the ms, or briefly explain why the item is not applicable/relevant for your study

In the intervention section of the manuscript: "Lose It! is a free for use mobile application."

5-viii) Mode of delivery, features/functionalities/components of the intervention and comparator, and the theoretical framework

Describe mode of delivery, features/functionalities/components of the intervention and comparator, and the theoretical framework [6] used to design them (instructional strategy [1], behaviour change techniques, persuasive features, etc., see e.g., [7, 8] for terminology). This includes an in-depth description of the content (including where it is coming from and who developed it) [1],” whether [and how] it is tailored to individual circumstances and allows users to track their progress and receive feedback” [6]. This also includes a description of communication delivery channels and – if computer-mediated communication is a component – whether communication was synchronous or asynchronous [6]. It also includes information on presentation strategies [1], including page design principles, average amount of text on pages, presence of hyperlinks to other resources, etc. [1].

	1	2	3	4	5	
subitem not at all important	○	○	○	◉	○	essential

Does your paper address subitem 5-viii? *

Copy and paste relevant sections from the manuscript (include quotes in quotation marks "like this" to indicate direct quotes from your manuscript), or elaborate on this item by providing additional information not in the ms, or briefly explain why the item is not applicable/relevant for your study

The Intervention section of the manuscript:

"SmartPhone Group
Participants were screened by the Stroke Social Worker and a member of the SOS research team during the acute hospitalization. After informed consent was obtained, the baseline clinic visit occurred within 2 weeks of hospital discharge.
During the baseline visit, a user account for the mobile application (Lose It! Version 5.2.1, released 03/07/2015, www.loseit.com) was created using the participant's e-mail address. Each participant and caregiver (if applicable) received a tutorial on how to download and use the Lose It! on his or her personally owned SmartPhone. Lose It! is a free for use mobile application.

For each participant, a ten percent weight loss goal was implemented, and a value for maximum daily caloric intake was provided under the "budget" column (Figure 1). SmartPhone group participants could search for foods, including restaurants, or scan a barcode, which uploaded the food product directly onto the Lose It! platform.
The number of calories consumed could be increased with physical activity, logged in the "exercise" column. The "net" calories each day equaled the total of food minus exercise. All SOS participants were provided premium features free of charge for six months, including the food journal group, after the study period. Details of the SmartPhone intervention protocol have been published [ ].

During the Initiation Phase (Baseline to Day 30), a pattern of compliance with Lose It! use was initiated. Participants received daily monitoring of caloric intake, with reminder messages via push notifications through the mobile application. We anticipated establishment of self-monitoring patterns during the Application Phase (Day 31 to 90). To facilitate positive reinforcement, the SmartPhone group received weekly push notification summaries of compliance, and reminder messages on missed days. The Maintenance Phase 3 (Day 91 to 180) consisted of weekly summaries with no reminder messages.

Food Journal Group
Similar to the SmartPhone group, a goal ten percent body weight reduction was calculated during the baseline clinic visit. To facilitate culturally competent care, dietary counseling was provided by a physician from the participants' ethnic group. The food journal group also received educational materials on a heart healthy diet from the American Heart Association (AHA) website and a paper food journal.

Interventions for Both Groups
Both the SmartPhone and food journal group received measuring cups, AHA cookbooks

Both the SmartPhone and food journal group received measuring cups, AHA cookbooks tailored for African-Americans or Hispanic populations, the US Department of Health and Human Services DASH Eating Plan guide and AHA reading materials (Suggested Servings from Each Food Group, Choosing a Restaurant, Dining Out Tips by Cuisine and Ordering your Meal). At the end of each phase, both groups returned to clinic for weight measurements and counseling. Study visits were at no cost to participants and caregivers."

5-ix) Describe use parameters

Describe use parameters (e.g., intended “doses” and optimal timing for use). Clarify what instructions or recommendations were given to the user, e.g., regarding timing, frequency, heaviness of use, if any, or was the intervention used ad libitum.

	1	2	3	4	5	
subitem not at all important	○	○	○	◉	○	essential

Does your paper address subitem 5-ix?

Copy and paste relevant sections from the manuscript (include quotes in quotation marks "like this" to indicate direct quotes from your manuscript), or elaborate on this item by providing additional information not in the ms, or briefly explain why the item is not applicable/relevant for your study

The feasibility and treatment fidelity Section of the manuscript:

"Feasibility and Treatment Fidelity
Study feasibility was measured using retention rates at Day 180 and adherence to the self-monitoring intervention at Day 30, 90 and 180. Compliance with the SmartPhone intervention was measured through a coaching tool integrated with the Lose It! platform (Ascend for Lose It!). Participants, with caregiver assistance, were instructed to enter food daily, either though the functionality of the mobile application or free text. Compliance with the food journal intervention was measured at the end of each phase through review of written entries. Caregivers were available to assist with food journal entries. Exit surveys were conducted at the end of the study period to learn about perceptions of the intervention."

5-x) Clarify the level of human involvement

Clarify the level of human involvement (care providers or health professionals, also technical assistance) in the e-intervention or as co-intervention (detail number and expertise of professionals involved, if any, as well as “type of assistance offered, the timing and frequency of the support, how it is initiated, and the medium by which the assistance is delivered”. It may be necessary to distinguish between the level of human involvement required for the trial, and the level of human involvement required for a routine application outside of a RCT setting (discuss under item 21 – generalizability).

	1	2	3	4	5	
subitem not at all important	○	○	○	○	◉	essential

Does your paper address subitem 5-x?

Copy and paste relevant sections from the manuscript (include quotes in quotation marks "like this" to indicate direct quotes from your manuscript), or elaborate on this item by providing additional information not in the ms, or briefly explain why the item is not applicable/relevant for your study

Yes, we have addressed this in both the Intervention and Feasibility and Treatment Fidelity sections of the manuscript:

"Intervention
SmartPhone Group
During the baseline visit, a user account for the mobile application (Lose It! Version 5.2.1, released 03/07/2015, www.loseit.com) was created using the participant's e-mail address. Each participant and caregiver (if applicable) received a tutorial on how to download and use the Lose It! on his or her personally owned SmartPhone. Lose It! is a free for use mobile application.
For each participant, a ten percent weight loss goal was implemented, and a value for maximum daily caloric intake was provided under the "budget" column (Figure 1). SmartPhone group participants could search for foods, including restaurants, or scan a barcode, which uploaded the food product directly onto the Lose It! platform.
The number of calories consumed could be increased with physical activity, logged in the "exercise" column. The "net" calories each day equaled the total of food minus exercise. All SOS participants were provided premium features free of charge for six months, including the food journal group, after the study period. Details of the SmartPhone intervention protocol have been published [ ].

During the Initiation Phase (Baseline to Day 30), a pattern of compliance with Lose It! use was initiated. Participants received daily monitoring of caloric intake, with reminder messages via push notifications through the mobile application. We anticipated establishment of self-monitoring patterns during the Application Phase (Day 31 to 90). To facilitate positive reinforcement, the SmartPhone group received weekly push notification summaries of compliance, and reminder messages on missed days. The Maintenance Phase 3 (Day 91 to 180) consisted of weekly summaries with no reminder messages.

Food Journal Group
Similar to the SmartPhone group, a goal ten percent body weight reduction was calculated during the baseline clinic visit. To facilitate culturally competent care, dietary counseling was provided by a physician from the participants' ethnic group. The food journal group also received educational materials on a heart healthy diet from the American Heart Association (AHA) website and a paper food journal.

Interventions for Both Groups
Both the SmartPhone and food journal group received measuring cups, AHA cookbooks tailored for African-Americans or Hispanic populations, the US Department of Health and Human Services DASH Eating Plan guide and AHA reading materials (Suggested Servings from Each Food Group, Choosing a Restaurant, Dining Out Tips by Cuisine and Ordering your Meal). At the end of each phase, both groups returned to clinic for weight measurements and counseling. Study visits were at no cost to participants and caregivers.

Feasibility and Treatment Fidelity
Study feasibility was measured using retention rates at Day 180 and adherence to the self-monitoring intervention at Day 30, 90 and 180. Compliance with the SmartPhone intervention was measured through a coaching tool integrated with the Lose It! platform (Ascend for Lose It!). Participants, with caregiver assistance, were instructed to enter food daily, either though the functionality of the mobile application or free text. Compliance with the food journal intervention was measured at the end of each phase through review of written entries. Caregivers were available to assist with food journal entries. Exit surveys were conducted at the end of the study period to learn about perceptions of the intervention."

5-xi) Report any prompts/reminders used

Report any prompts/reminders used: Clarify if there were prompts (letters, emails, phone calls, SMS) to use the application, what triggered them, frequency etc. It may be necessary to distinguish between the level of prompts/reminders required for the trial, and the level of prompts/reminders for a routine application outside of a RCT setting (discuss under item 21 – generalizability).

	1	2	3	4	5	
subitem not at all important	○	○	○	○	◉	essential

Does your paper address subitem 5-xi? *

Copy and paste relevant sections from the manuscript (include quotes in quotation marks "like this" to indicate direct quotes from your manuscript), or elaborate on this item by providing additional information not in the ms, or briefly explain why the item is not applicable/relevant for your study

"During the Initiation Phase (Baseline to Day 30), a pattern of compliance with Lose It! use was initiated. Participants received daily monitoring of caloric intake, with reminder messages via push notifications through the mobile application. We anticipated establishment of self-monitoring patterns during the Application Phase (Day 31 to 90). To facilitate positive reinforcement, the SmartPhone group received weekly push notification summaries of compliance, and reminder messages on missed days. The Maintenance Phase 3 (Day 91 to 180) consisted of weekly summaries with no reminder messages."

We have also added a sentence referring readers to the protocol publication, in which the prompts and reminder structure is given in detail:
The publication is: Ifejika NL, Noser EA, Grotta JC, Savitz SI. Swipe out Stroke: Feasibility and efficacy of using a smart-phone based mobile application to improve compliance with weight loss in obese minority stroke patients and their carers. Int J Stroke. 2016;11:593-603.

5-xii) Describe any co-interventions (incl. training/support)

Describe any co-interventions (incl. training/support): Clearly state any interventions that are provided in addition to the targeted eHealth intervention, as ehealth intervention may not be designed as stand-alone intervention. This includes training sessions and support [1]. It may be necessary to distinguish between the level of training required for the trial, and the level of training for a routine application outside of a RCT setting (discuss under item 21 – generalizability.

	1	2	3	4	5	
subitem not at all important	(•)	( )	( )	( )	( )	essential

Does your paper address subitem 5-xii? *

Copy and paste relevant sections from the manuscript (include quotes in quotation marks "like this" to indicate direct quotes from your manuscript), or elaborate on this item by providing additional information not in the ms, or briefly explain why the item is not applicable/relevant for your study

There was no co-intervention in addition to the eHealth intervention.

6a) Completely defined pre-specified primary and secondary outcome measures, including how and when they were assessed

Does your paper address CONSORT subitem 6a? *

Copy and paste relevant sections from the manuscript (include quotes in quotation marks "like this" to indicate direct quotes from your manuscript), or elaborate on this item by providing additional information not in the ms, or briefly explain why the item is not applicable/relevant for your study

"The primary outcome was a reduction in total body weight. The United States Preventative Services Task Force (USPSTF) guidelines indicate a weight reduction of 5 to 10 percent yields measurable improvement in risk factors for cerebrovascular disease [ ]. We measured overall weight reduction in both the SmartPhone and food journal groups. Secondary outcomes included compliance with the weight loss intervention, improvement in depression and, if abnormal, normalization of systolic blood pressure, serum low density lipoprotein value, serum hemoglobin A1c and percentage of serum Factor VIII."

6a-i) Online questionnaires: describe if they were validated for online use and apply CHERRIES items to describe how the questionnaires were designed/deployed

If outcomes were obtained through online questionnaires, describe if they were validated for online use and apply CHERRIES items to describe how the questionnaires were designed/deployed [9].

	1	2	3	4	5	
subitem not at all important	(●)	( )	( )	( )	( )	essential

Does your paper address subitem 6a-i?

Copy and paste relevant sections from manuscript text

We did not use online questionnaires to measure study outcomes.

6a-ii) Describe whether and how “use” (including intensity of use/dosage) was defined/measured/monitored

Describe whether and how “use” (including intensity of use/dosage) was defined/measured/monitored (logins, logfile analysis, etc.). Use/adoption metrics are important process outcomes that should be reported in any ehealth trial.

	1	2	3	4	5	
subitem not at all important	( )	( )	( )	( )	( )	essential

Does your paper address subitem 6a-ii?

Copy and paste relevant sections from manuscript text

" Compliance with the SmartPhone intervention was measured through a coaching tool integrated with the Lose It! platform (Ascend for Lose It!). Participants, with caregiver assistance, were instructed to enter food daily, either though the functionality of the mobile application or free text. Compliance with the food journal intervention was measured at the end of each phase through review of written entries. Caregivers were available to assist with food journal entries."

6a-iii) Describe whether, how, and when qualitative feedback from participants was obtained

Describe whether, how, and when qualitative feedback from participants was obtained (e.g., through emails, feedback forms, interviews, focus groups).

	1	2	3	4	5	
subitem not at all important	○	○	●	○	○	essential

Does your paper address subitem 6a-iii?

Copy and paste relevant sections from manuscript text

From the methods section:
"We used a mixed method design with quantitative measures. Exit surveys were distributed to determine participants' acceptance of the intervention; the quantitative results are reported in this paper."

From the discussion section:
"Study participants used their personally owned SmartPhones and during exit interviews, several participants noted financial limitations during the study period. The adherence to self-monitoring may be different if SmartPhones with data plans were provided."

6b) Any changes to trial outcomes after the trial commenced, with reasons

Does your paper address CONSORT subitem 6b? *

Copy and paste relevant sections from the manuscript (include quotes in quotation marks "like this" to indicate direct quotes from your manuscript), or elaborate on this item by providing additional information not in the ms, or briefly explain why the item is not applicable/relevant for your study

There were no changes to the trial outcomes after the study commenced.

7a) How sample size was determined

NPT: When applicable, details of whether and how the clustering by care provides or centers was addressed

7a-i) Describe whether and how expected attrition was taken into account when calculating the sample size

Describe whether and how expected attrition was taken into account when calculating the sample size.

	1	2	3	4	5	
subitem not at all important	○	◉	○	○	○	essential

Does your paper address subitem 7a-i?

Copy and paste relevant sections from manuscript title (include quotes in quotation marks "like this" to indicate direct quotes from your manuscript), or elaborate on this item by providing additional information not in the ms, or briefly explain why the item is not applicable/relevant for your study

This was a pilot study - we aimed to recruit 50 participants, with a goal of 15 in each group. During the study period (March 2015 to December 2016), the Houston area had several floods and a large hurricane, many of our participants were affected.
We recruited 36 participants (17 in the SmartPhone group, 19 in the food journal group) and retained 25 total. In a minority only pilot study of stroke survivors in this setting, we were very much encouraged by our retention rate.

7b) When applicable, explanation of any interim analyses and stopping guidelines

Does your paper address CONSORT subitem 7b? *

Copy and paste relevant sections from the manuscript (include quotes in quotation marks "like this" to indicate direct quotes from your manuscript), or elaborate on this item by providing additional information not in the ms, or briefly explain why the item is not applicable/relevant for your study

There was no interim analysis. The study did not stop at any time period.

8a) Method used to generate the random allocation sequence

NPT: When applicable, how care providers were allocated to each trial group

Does your paper address CONSORT subitem 8a? *

Copy and paste relevant sections from the manuscript (include quotes in quotation marks "like this" to indicate direct quotes from your manuscript), or elaborate on this item by providing additional information not in the ms, or briefly explain why the item is not applicable/relevant for your study

There was no random allocation sequence.

"To facilitate culturally competent care, dietary counseling was provided by a physician from the participants’ ethnic group."

8b) Type of randomisation; details of any restriction (such as blocking and block size)

Does your paper address CONSORT subitem 8b? *

Copy and paste relevant sections from the manuscript (include quotes in quotation marks "like this" to indicate direct quotes from your manuscript), or elaborate on this item by providing additional information not in the ms, or briefly explain why the item is not applicable/relevant for your study

"Randomization

Stroke survivors were randomized to the SmartPhone group or food journal group in a 1:1 ratio, using an adaptive covariate randomization algorithm. The adaptive randomization schema utilized Pearson's $\chi 2$ statistic to measure treatment imbalances in stroke severity (Modified Rankin Scale of 0 to 1 : no symptoms to no significant disability or modified Rankin Scale of 2 to 3: slight to moderate disability), age and sex. The presence of depression was determined at randomization using the Patient Health Questionnaire-9 (PHQ-9) survey, and was equally allocated to the SmartPhone and food journal groups in three categories – zero to minimal (0 to 4) , mild to moderate (5 to 14) and moderately severe to severe (>14)."

9) Mechanism used to implement the random allocation sequence (such as sequentially numbered containers), describing any steps taken to conceal the sequence until interventions were assigned

Does your paper address CONSORT subitem 9? *

Copy and paste relevant sections from the manuscript (include quotes in quotation marks "like this" to indicate direct quotes from your manuscript), or elaborate on this item by providing additional information not in the ms, or briefly explain why the item is not applicable/relevant for your study

"Each new subject was randomly assigned to the group that achieved the best treatment balance, using a web-based data management application to generate the random allocation sequence."

10) Who generated the random allocation sequence, who enrolled participants, and who assigned participants to interventions

Does your paper address CONSORT subitem 10? *

Copy and paste relevant sections from the manuscript (include quotes in quotation marks "like this" to indicate direct quotes from your manuscript), or elaborate on this item by providing additional information not in the ms, or briefly explain why the item is not applicable/relevant for your study

A web based data management application generated the random allocation sequence that assigned participants to either the SmartPhone or Food Journal group.

Screening of participants:
"Participants were screened by the Stroke Social Worker and a member of the SOS research team during the acute hospitalization. After informed consent was obtained, the baseline clinic visit occurred within 2 weeks of hospital discharge."

Interventions for Both Groups:
"Participants in both groups received dietary counseling by a study team member of their racial background at each visit (African-American or Hispanic)."

11a) If done, who was blinded after assignment to interventions (for example, participants, care providers, those assessing outcomes) and how

NPT: Whether or not administering co-interventions were blinded to group assignment

11a-i) Specify who was blinded, and who wasn’t

Specify who was blinded, and who wasn’t. Usually, in web-based trials it is not possible to blind the participants [1, 3] (this should be clearly acknowledged), but it may be possible to blind outcome assessors, those doing data analysis or those administering co-interventions (if any).

	1	2	3	4	5	
subitem not at all important	○	◉	○	○	○	essential

Does your paper address subitem 11a-i? *

Copy and paste relevant sections from the manuscript (include quotes in quotation marks "like this" to indicate direct quotes from your manuscript), or elaborate on this item by providing additional information not in the ms, or briefly explain why the item is not applicable/relevant for your study

This study was a Prospective randomized control trial with an open blinded endpoint (PROBE).
In the study design section:
"The blinded end point committee consisted of the senior authors (Grotta, Savitz) and the statistician (Cai)."

11a-ii) Discuss e.g., whether participants knew which intervention was the "intervention of interest" and which one was the "comparator"

Informed consent procedures (4a-ii) can create biases and certain expectations - discuss e.g., whether participants knew which intervention was the "intervention of interest" and which one was the "comparator".

	1	2	3	4	5	
subitem not at all important	○	○	○	◉	○	essential

Does your paper address subitem 11a-ii?

Copy and paste relevant sections from the manuscript (include quotes in quotation marks "like this" to indicate direct quotes from your manuscript), or elaborate on this item by providing additional information not in the ms, or briefly explain why the item is not applicable/relevant for your study

All participants received in person visits at baseline, Day 30, 90 and 180. The informed consent document detailed differences between the food journal and SmartPhone groups regarding the duration and frequency of additional SmartPhone engagement.

11b) If relevant, description of the similarity of interventions

(this item is usually not relevant for ehealth trials as it refers to similarity of a placebo or sham intervention to a active medication/intervention)

Does your paper address CONSORT subitem 11b? *

Copy and paste relevant sections from the manuscript (include quotes in quotation marks "like this" to indicate direct quotes from your manuscript), or elaborate on this item by providing additional information not in the ms, or briefly explain why the item is not applicable/relevant for your study

Yes, differences between the SmartPhone and Food Journal intervention are detailed below.

"Intervention

SmartPhone Group
During the baseline visit, a user account for the mobile application(Lose It! Version 5.2.1, released 03/07/2015, www.loseit.com) was created by a member of the study team using the participant's e-mail address. Each participant and caregiver (if applicable) received a tutorial on how to download and use the Lose It! on his or her personally owned SmartPhone. Lose It! is a free for use mobile application.
For each participant, a ten percent weight loss goal was implemented, and a value for maximum daily caloric intake was provided under the "budget" column (Figure 1). SmartPhone group participants could search for foods, including restaurants, or scan a barcode, which uploaded the food product directly onto the Lose It! platform.
The number of calories consumed could be increased with physical activity, logged in the "exercise" column. The "net" calories each day equaled the total of food minus exercise. All SOS participants were provided premium features free of charge for six months, including the food journal group, after the study period. Details of the SmartPhone intervention protocol have been published [ ].

During the Initiation Phase (Baseline to Day 30), a pattern of compliance with Lose It! use was initiated. Participants received daily monitoring of caloric intake, with reminder messages via push notifications through the mobile application. We anticipated establishment of self-monitoring patterns during the Application Phase (Day 31 to 90). To facilitate positive reinforcement, the SmartPhone group received weekly push notification summaries of compliance, and reminder messages on missed days. The Maintenance Phase 3 (Day 91 to 180) consisted of weekly summaries with no reminder messages. Examples of each messages sent during each phase are detailed in a previous publication [18].

Food Journal Group
Similar to the SmartPhone group, a goal ten percent body weight reduction was calculated during the baseline clinic visit. To facilitate culturally competent care, dietary counseling was provided by a physician from the participants' ethnic group. The food journal group also received educational materials on a heart healthy diet from the American Heart Association (AHA) website and a paper food journal. "

12a) Statistical methods used to compare groups for primary and secondary outcomes

NPT: When applicable, details of whether and how the clustering by care providers or centers was addressed

Does your paper address CONSORT subitem 12a? *

Copy and paste relevant sections from the manuscript (include quotes in quotation marks "like this" to indicate direct quotes from your manuscript), or elaborate on this item by providing additional information not in the ms, or briefly explain why the item is not applicable/relevant for your study

Yes - the statistical analysis section of the manuscript:

"Descriptive statistics (frequency and percentage for categorical variables and mean and standard deviation / median and interquartile range for continuous variables) were reported. Two sample t-test or Wilcoxon rank-sum test were used to compare continuous variables; Fisher's exact test was used to compare categorical variables.
Demographics and self-reported cardiovascular risk factors were obtained by self-report. Cognitive testing was completed at baseline; depression screening was completed at baseline, Days 30, 90 and 180, and compared between the SmartPhone and food journal groups using Wilcoxon rank sum test. The primary outcome was measured as weight change in pounds; weight change was also compared by the presence of depression (defined as self-reported depression or PHQ-9 value ≥ 5). All analyses were performed using SAS 9.4 (Cary, NC); P<0.05 was considered significant."

12a-i) Imputation techniques to deal with attrition / missing values

Imputation techniques to deal with attrition / missing values: Not all participants will use the intervention/comparator as intended and attrition is typically high in ehealth trials. Specify how participants who did not use the application or dropped out from the trial were treated in the statistical analysis (a complete case analysis is strongly discouraged, and simple imputation techniques such as LOCF may also be problematic [4]).

	1	2	3	4	5	
subitem not at all important	○	○	○	○	◉	essential

Does your paper address subitem 12a-i? *

Copy and paste relevant sections from the manuscript (include quotes in quotation marks "like this" to indicate direct quotes from your manuscript), or elaborate on this item by providing additional information not in the ms, or briefly explain why the item is not applicable/relevant for your study

We did not impute data. These values listed were obtained at the study time points.

12b) Methods for additional analyses, such as subgroup analyses and adjusted analyses

Does your paper address CONSORT subitem 12b? *

Copy and paste relevant sections from the manuscript (include quotes in quotation marks "like this" to indicate direct quotes from your manuscript), or elaborate on this item by providing additional information not in the ms, or briefly explain why the item is not applicable/relevant for your study

Yes - we did a subgroup analysis of weight by depression status:
"Weight change was also compared by the presence of depression (defined as self-reported depression or PHQ-9 value ≥ 5)."

We avoided doing multiple subgroup analyses in this pilot population due to the lack of appropriate power to yield a true difference.

X26) REB/IRB Approval and Ethical Considerations [recommended as subheading under "Methods"] (not a CONSORT item)

X26-i) Comment on ethics committee approval

	1	2	3	4	5	
subitem not at all important	○	○	○	○	◉	essential

Does your paper address subitem X26-i?

Copy and paste relevant sections from the manuscript (include quotes in quotation marks "like this" to indicate direct quotes from your manuscript), or elaborate on this item by providing additional information not in the ms, or briefly explain why the item is not applicable/relevant for your study

"This study was approved by the Institutional Review Board at the McGovern Medical School at UTHealth (HSC-MS-13-0608)."

x26-ii) Outline informed consent procedures

Outline informed consent procedures e.g., if consent was obtained offline or online (how? Checkbox, etc.?), and what information was provided (see 4a-ii). See [6] for some items to be included in informed consent documents.

	1	2	3	4	5	
subitem not at all important	○	○	○	○	◉	essential

Does your paper address subitem X26-ii?

Copy and paste relevant sections from the manuscript (include quotes in quotation marks "like this" to indicate direct quotes from your manuscript), or elaborate on this item by providing additional information not in the ms, or briefly explain why the item is not applicable/relevant for your study

"This study was approved by the Institutional Review Board at the McGovern Medical School at UTHealth (HSC-MS-13-0608). Informed consent was obtained from both study participants and their caregivers, detailing the purpose of the study, frequency of visits, descriptors of the SmartPhone and food journal interventions, benefits, risks, protection of privacy and study withdrawal procedures."

X26-iii) Safety and security procedures

Safety and security procedures, incl. privacy considerations, and any steps taken to reduce the likelihood or detection of harm (e.g., education and training, availability of a hotline)

	1	2	3	4	5	
subitem not at all important	○	○	◉	○	○	essential

Does your paper address subitem X26-iii?

Copy and paste relevant sections from the manuscript (include quotes in quotation marks "like this" to indicate direct quotes from your manuscript), or elaborate on this item by providing additional information not in the ms, or briefly explain why the item is not applicable/relevant for your study

Privacy considerations were detailed in the informed consent and noted in the manuscript: "Informed consent was obtained from both study participants and their caregivers, detailing the purpose of the study, frequency of visits, descriptors of the SmartPhone and food journal interventions, benefits, risks, protection of privacy and study withdrawal procedures."

All study participants were obese stroke survivors, at a significant risk of recurrent stroke. An observational weight management intervention in this population has a limited likelihood of harm.

RESULTS

13a) For each group, the numbers of participants who were randomly assigned, received intended treatment, and were analysed for the primary outcome

NPT: The number of care providers or centers performing the intervention in each group and the number of patients treated by each care provider in each center

Does your paper address CONSORT subitem 13a? *

Copy and paste relevant sections from the manuscript (include quotes in quotation marks "like this" to indicate direct quotes from your manuscript), or elaborate on this item by providing additional information not in the ms, or briefly explain why the item is not applicable/relevant for your study

From the results section:

"Equal allocation was achieved between the SmartPhone (n=17) and food journal (n=19) groups."

"One patient died in the SmartPhone group. The overall retention rate was 77.7% (28/36) at Day 30, 72.2% (26/36) at Day 90 and 69.4% (25/36) at Day 180. The CONSORT diagram depicting patient retention is in Figure 2."

13b) For each group, losses and exclusions after randomisation, together with reasons

Does your paper address CONSORT subitem 13b? (NOTE: Preferably, this is shown in a CONSORT flow diagram) *

Copy and paste relevant sections from the manuscript (include quotes in quotation marks "like this" to indicate direct quotes from your manuscript), or elaborate on this item by providing additional information not in the ms, or briefly explain why the item is not applicable/relevant for your study

Figure 2 in the manuscript details the losses after randomization and reasons.

13b-i) Attrition diagram

Strongly recommended: An attrition diagram (e.g., proportion of participants still logging in or using the intervention/comparator in each group plotted over time, similar to a survival curve) or other figures or tables demonstrating usage/dose/engagement.

	1	2	3	4	5	
subitem not at all important	○	○	○	○	●	essential

Does your paper address subitem 13b-i?

Copy and paste relevant sections from the manuscript or cite the figure number if applicable (include quotes in quotation marks "like this" to indicate direct quotes from your manuscript), or elaborate on this item by providing additional information not in the ms, or briefly explain why the item is not applicable/relevant for your study

"Intervention adherence to one dietary entry per day was significantly higher at Day 90 in the SmartPhone group compared to the food journal group (58.8 vs 21.1%; P=0.04).
In the SmartPhone group, adherence to one self-monitored diet entry per day was 58.8% (10/17) at Day 30 and 35.3% (6/17) at Day 180.
Adherence to one self-monitored diet entry per day in the food journal group was 42.1% (8/19) at Day 30 and 10.5% (2/19) at Day 180.

14a) Dates defining the periods of recruitment and follow-up

Does your paper address CONSORT subitem 14a? *

Copy and paste relevant sections from the manuscript (include quotes in quotation marks "like this" to indicate direct quotes from your manuscript), or elaborate on this item by providing additional information not in the ms, or briefly explain why the item is not applicable/relevant for your study

"Participants were enrolled in Swipe out Stroke between March 2015 and May 2016. The final follow-up visit occurred in December 2016."

14a-i) Indicate if critical "secular events" fell into the study period

Indicate if critical "secular events" fell into the study period, e.g., significant changes in Internet resources available or "changes in computer hardware or Internet delivery resources"

	1	2	3	4	5	
subitem not at all important	○	○	◉	○	○	essential

Does your paper address subitem 14a-i?

Copy and paste relevant sections from the manuscript (include quotes in quotation marks "like this" to indicate direct quotes from your manuscript), or elaborate on this item by providing additional information not in the ms, or briefly explain why the item is not applicable/relevant for your study

"There were no significant changes in computer hardware or internet delivery resources during the study period."

14b) Why the trial ended or was stopped (early)

Does your paper address CONSORT subitem 14b? *

Copy and paste relevant sections from the manuscript (include quotes in quotation marks "like this" to indicate direct quotes from your manuscript), or elaborate on this item by providing additional information not in the ms, or briefly explain why the item is not applicable/relevant for your study

The trial ended upon reaching 15 participants in each group.

15) A table showing baseline demographic and clinical characteristics for each group

NPT: When applicable, a description of care providers (case volume, qualification, expertise, etc.) and centers (volume) in each group

Does your paper address CONSORT subitem 15? *

Copy and paste relevant sections from the manuscript (include quotes in quotation marks "like this" to indicate direct quotes from your manuscript), or elaborate on this item by providing additional information not in the ms, or briefly explain why the item is not applicable/relevant for your study

Table 2 lists demographic and clinical characteristics for each group.

15-i) Report demographics associated with digital divide issues

In ehealth trials it is particularly important to report demographics associated with digital divide issues, such as age, education, gender, social-economic status, computer/Internet/ehealth literacy of the participants, if known.

	1	2	3	4	5	
subitem not at all important	○	○	○	○	◉	essential

Does your paper address subitem 15-i? *

Copy and paste relevant sections from the manuscript (include quotes in quotation marks "like this" to indicate direct quotes from your manuscript), or elaborate on this item by providing additional information not in the ms, or briefly explain why the item is not applicable/relevant for your study

Age, Education, Gender and Employment Status are listed in Table 2.

16) For each group, number of participants (denominator) included in each analysis and whether the analysis was by original assigned groups

16-i) Report multiple “denominators” and provide definitions

Report multiple “denominators” and provide definitions: Report N’s (and effect sizes) “across a range of study participation [and use] thresholds” [1], e.g., N exposed, N consented, N used more than x times, N used more than y weeks, N participants “used” the intervention/comparator at specific pre-defined time points of interest (in absolute and relative numbers per group). Always clearly define “use” of the intervention.

	1	2	3	4	5	
subitem not at all important	○	○	○	○	◉	essential

Does your paper address subitem 16-i? *

Copy and paste relevant sections from the manuscript (include quotes in quotation marks "like this" to indicate direct quotes from your manuscript), or elaborate on this item by providing additional information not in the ms, or briefly explain why the item is not applicable/relevant for your study

Denominators and definitions are provided as detailed here:
"Sixty-nine percent of stroke survivors completed Swipe out Stroke (n=25, 13/17 in the SmartPhone group, 12/19 in the food journal group); one participant died in the SmartPhone group.
The overall retention rate was 77.7% (28/36) at Day 30, 72.2% (26/36) at Day 90 and 69.4% (25/36) at Day 180."

"Intervention adherence to one dietary entry per day was significantly higher at Day 90 in the SmartPhone group compared to the food journal group (58.8 vs 21.1%; P=0.04).
In the SmartPhone group, adherence to one self-monitored diet entry per day was 58.8% (10/17) at Day 30 and 35.3% (6/17) at Day 180.
Adherence to one self-monitored diet entry per day in the food journal group was 42.1% (8/19) at Day 30 and 10.5% (2/19) at Day 180."

16-ii) Primary analysis should be intent-to-treat

Primary analysis should be intent-to-treat, secondary analyses could include comparing only “users”, with the appropriate caveats that this is no longer a randomized sample (see 18-i).

	1	2	3	4	5	
subitem not at all important	(•)	( )	( )	( )	( )	essential

Does your paper address subitem 16-ii?

Copy and paste relevant sections from the manuscript (include quotes in quotation marks "like this" to indicate direct quotes from your manuscript), or elaborate on this item by providing additional information not in the ms, or briefly explain why the item is not applicable/relevant for your study

This pilot study was not powered to detect differences. After rigorous discussion with the study team, it was determined that a subgroup analysis in 8 out of 25 participants would not be reliable, and in an understudied population (minority stroke survivors within the first six months of event), may not be advisable.

17a) For each primary and secondary outcome, results for each group, and the estimated effect size and its precision (such as 95% confidence interval)

Does your paper address CONSORT subitem 17a? *

Copy and paste relevant sections from the manuscript (include quotes in quotation marks "like this" to indicate direct quotes from your manuscript), or elaborate on this item by providing additional information not in the ms, or briefly explain why the item is not applicable/relevant for your study

"Descriptive findings on weight outcomes at each study collection time point (Day 30, Day 90, Day 180) are summarized in Figure 3. There were no statistically significant differences in weight loss between the SmartPhone and food journal groups.
At Day 180, the SmartPhone group had a median weight loss of 5.7 pounds (IQR -2.4 to 8.0). Median weight loss for the food journal group was 6.4 pounds (IQR -2.2 to 12.5; P=0.77). There was a 2.1% median weight change in the SmartPhone group, compared to a 2.9% median weight change in the usual care group (P=0.63).
At Day 180, study participants with depression sustained a median weight loss of 3.9 pounds (IQR -10.1 to 14), participants without depression lost 6.4 pounds (IQR -2.4 to 15; P=0.49)."

17a-i) Presentation of process outcomes such as metrics of use and intensity of use

In addition to primary/secondary (clinical) outcomes, the presentation of process outcomes such as metrics of use and intensity of use (dose, exposure) and their operational definitions is critical. This does not only refer to metrics of attrition (13-b) (often a binary variable), but also to more continuous exposure metrics such as “average session length”. These must be accompanied by a technical description how a metric like a “session” is defined (e.g., timeout after idle time) [1] (report under item 6a).

	1	2	3	4	5	
subitem not at all important	○	○	○	●	○	essential

Does your paper address subitem 17a-i?

Copy and paste relevant sections from the manuscript (include quotes in quotation marks "like this" to indicate direct quotes from your manuscript), or elaborate on this item by providing additional information not in the ms, or briefly explain why the item is not applicable/relevant for your study

SmartPhone portion of the intervention section:
"SmartPhone engagement by a member of the study team took approximately 3 minutes per participant during the Initiation Phase, 2 minutes per participant during the Application Phase and one minute per participant during the Maintenance Phase. "

Interventions for Both Groups portion of the Intervention section:
"The duration of each in person study visit was 30 minutes, study visits were at no cost to participants and caregivers."

In the results section:
"Intervention adherence to one dietary entry per day was significantly higher at Day 90 in the SmartPhone group compared to the food journal group (58.8 vs 21.1%; P=0.04).
In the SmartPhone group, adherence to one self-monitored diet entry per day was 58.8% (10/17) at Day 30 and 35.3% (6/17) at Day 180.
Adherence to one self-monitored diet entry per day in the food journal group was 42.1% (8/19) at Day 30 and 10.5% (2/19) at Day 180."

17b) For binary outcomes, presentation of both absolute and relative effect sizes is recommended

Does your paper address CONSORT subitem 17b? *

Copy and paste relevant sections from the manuscript (include quotes in quotation marks "like this" to indicate direct quotes from your manuscript), or elaborate on this item by providing additional information not in the ms, or briefly explain why the item is not applicable/relevant for your study

We did not look at binary outcomes in this study.

18) Results of any other analyses performed, including subgroup analyses and adjusted analyses, distinguishing pre-specified from exploratory

Does your paper address CONSORT subitem 18? *

Copy and paste relevant sections from the manuscript (include quotes in quotation marks "like this" to indicate direct quotes from your manuscript), or elaborate on this item by providing additional information not in the ms, or briefly explain why the item is not applicable/relevant for your study

Figure 3 illustrates overall weight loss and weight loss in patients who screened positive for depression.
In the results section:
"At Day 180, study participants with depression sustained a median weight loss of 3.9 pounds (IQR -10.1 to 14), participants without depression lost 6.4 pounds (IQR -2.4 to 15; P=0.49)."

Figure 4 details the presence of depression in the SmartPhone versus Food Journal groups.
We completed a subgroup analysis in patients who screened positive for depression:
Depression was significantly lower at 30 days in the SmartPhone group compared to the food journal group (PHQ-9 of 2 vs 8; P=0.03)(Figure 4).
Clinically relevant depression rates remained in the “zero to minimal” range for the SmartPhone group compared to “mild to moderate” range in the food journal group at Day 90 (PHQ-9 of 3.5 vs 4.5; P=0.39) and Day 180 (PHQ-9 of 3 vs 6; P=0.12) (Figure 4).

18-i) Subgroup analysis of comparing only users

A subgroup analysis of comparing only users is not uncommon in ehealth trials, but if done, it must be stressed that this is a self-selected sample and no longer an unbiased sample from a randomized trial (see 16-iii).

	1	2	3	4	5	
subitem not at all important	(selected)	○	○	○	○	essential

Does your paper address subitem 18-i?

Copy and paste relevant sections from the manuscript (include quotes in quotation marks "like this" to indicate direct quotes from your manuscript), or elaborate on this item by providing additional information not in the ms, or briefly explain why the item is not applicable/relevant for your study

After rigorous discussion with the study team, it was determined that a subgroup analysis in 8 out of 25 participants would not be reliable, and in an understudied population (minority stroke survivors within the first six months of event), may not be advisable.

19) All important harms or unintended effects in each group

(for specific guidance see CONSORT for harms)

Does your paper address CONSORT subitem 19? *

Copy and paste relevant sections from the manuscript (include quotes in quotation marks "like this" to indicate direct quotes from your manuscript), or elaborate on this item by providing additional information not in the ms, or briefly explain why the item is not applicable/relevant for your study

Yes, in the discussion section:

"We found significantly decreased depression rates in the SmartPhone group at 30 days, which remained in the zero to minimal range; the food journal group had PHQ-9 scores indicative of mild to moderate depression throughout this study. Although both groups had built in caregiver support, the SmartPhone group received reminder messages and positive reinforcement on a daily, then weekly basis, providing support for the weight loss intervention. During the first six months post-stroke, depression appears to be reactive, and is correlated with increased severity of impairment in activities of daily living at one year [ ]. The effect of direct patient engagement via mobile applications, on both post-stroke depression and weight loss, is an exciting direction of future studies."

19-i) Include privacy breaches, technical problems

Include privacy breaches, technical problems. This does not only include physical “harm” to participants, but also incidents such as perceived or real privacy breaches [1], technical problems, and other unexpected/unintended incidents. “Unintended effects” also includes unintended positive effects [2].

	1	2	3	4	5	
subitem not at all important	○	○	○	◉	○	essential

Does your paper address subitem 19-i?

Copy and paste relevant sections from the manuscript (include quotes in quotation marks "like this" to indicate direct quotes from your manuscript), or elaborate on this item by providing additional information not in the ms, or briefly explain why the item is not applicable/relevant for your study

No technical problems, unintended positive effects were noted in the SmartPhone group:

"We found significantly decreased depression rates in the SmartPhone group at 30 days, which remained in the zero to minimal range; the food journal group had PHQ-9 scores indicative of mild to moderate depression throughout this study. Although both groups had built in caregiver support, the SmartPhone group received reminder messages and positive reinforcement on a daily, then weekly basis, providing support for the weight loss intervention. The effect of direct patient engagement via mobile applications, on both post-stroke depression and weight loss, is an exciting direction of future studies."

19-ii) Include qualitative feedback from participants or observations from staff/researchers

Include qualitative feedback from participants or observations from staff/researchers, if available, on strengths and shortcomings of the application, especially if they point to unintended/unexpected effects or uses. This includes (if available) reasons for why people did or did not use the application as intended by the developers.

	1	2	3	4	5	
subitem not at all important	○	○	◉	○	○	essential

Does your paper address subitem 19-ii?

Copy and paste relevant sections from the manuscript (include quotes in quotation marks "like this" to indicate direct quotes from your manuscript), or elaborate on this item by providing additional information not in the ms, or briefly explain why the item is not applicable/relevant for your study

"Study participants used their personally owned SmartPhones and during exit interviews, several participants noted financial limitations during the study period. The adherence to self-monitoring may be different if SmartPhones with data plans were provided."

DISCUSSION

22) Interpretation consistent with results, balancing benefits and harms, and considering other relevant evidence

NPT: In addition, take into account the choice of the comparator, lack of or partial blinding, and unequal expertise of care providers or centers in each group

22-i) Restate study questions and summarize the answers suggested by the data, starting with primary outcomes and process outcomes (use)

Restate study questions and summarize the answers suggested by the data, starting with primary outcomes and process outcomes (use).

	1	2	3	4	5	
subitem not at all important	○	○	○	○	◉	essential

Does your paper address subitem 22-i? *

Copy and paste relevant sections from the manuscript (include quotes in quotation marks "like this" to indicate direct quotes from your manuscript), or elaborate on this item by providing additional information not in the ms, or briefly explain why the item is not applicable/relevant for your study

"We compared the feasibility and preliminary treatment effects of a standard behavioral lifestyle intervention model using food journals to a mobile application platform with positive reinforcement messages to improve weight management in an ethnically diverse community. To our knowledge, this study is the first to implement a self-monitored SmartPhone based intervention, with caregiver support, in patients with post-stroke cognitive impairments. The time interval immediately following an acute stroke presents the opportunity to successfully change dietary patterns in patients and their caregivers, who influence the availability of healthy food choices.
The overall retention rates are promising, and establish an early precedent in this population at increased risk for worsening cerebrovascular disease and other vascular comorbidities.
The comparative findings of increased patient engagement and adherence to self-monitoring in the SmartPhone group are consistent with previous studies reporting higher adherence to self-monitoring rates using electronic versus paper food diaries among overweight or obese populations."

22-ii) Highlight unanswered new questions, suggest future research

Highlight unanswered new questions, suggest future research.

	1	2	3	4	5	
subitem not at all important	○	○	○	●	○	essential

Does your paper address subitem 22-ii?

Copy and paste relevant sections from the manuscript (include quotes in quotation marks "like this" to indicate direct quotes from your manuscript), or elaborate on this item by providing additional information not in the ms, or briefly explain why the item is not applicable/relevant for your study

"We found significantly decreased depression rates in the SmartPhone group at 30 days, which remained in the zero to minimal range; the food journal group had PHQ-9 scores indicative of mild to moderate depression throughout this study. Although both groups had built in caregiver support, the SmartPhone group received reminder messages and positive reinforcement on a daily, then weekly basis, providing support for the weight loss intervention. During the first six months post-stroke, depression appears to be reactive, and is correlated with increased severity of impairment in activities of daily living at one year [ ]. The effect of direct patient engagement via mobile applications, on both post-stroke depression and weight loss, is an exciting direction of future studies.
There is a known relationship between post-stroke depression and cognitive impairment [32]. Several studies demonstrated lower Mini-Mental Status Examination scores in patients with major depression when matched with non-depressed patients with similar stroke volume and stroke location [,]. Participants in this study screened positive for mild to moderate depression at randomization; we counseled all participants to follow-up with their primary care provider regarding initiation of treatment. Furthermore, we endeavored to improve study compliance by including caregivers in the protocol."

20) Trial limitations, addressing sources of potential bias, imprecision, and, if relevant, multiplicity of analyses

20-i) Typical limitations in ehealth trials

Typical limitations in ehealth trials: Participants in ehealth trials are rarely blinded. Ehealth trials often look at a multiplicity of outcomes, increasing risk for a Type I error. Discuss biases due to non-use of the intervention/usability issues, biases through informed consent procedures, unexpected events.

	1	2	3	4	5	
subitem not at all important	○	○	○	◉	○	essential

Does your paper address subitem 20-i? *

Copy and paste relevant sections from the manuscript (include quotes in quotation marks "like this" to indicate direct quotes from your manuscript), or elaborate on this item by providing additional information not in the ms, or briefly explain why the item is not applicable/relevant for your study

"There are several limitations to this study. The primary focus of this pilot study was feasibility and early treatment effect; there was not sufficient power to detect group differences. Study participants used their personally owned SmartPhones and during exit interviews, several participants noted financial limitations during the study period. The adherence to self-monitoring may be different if SmartPhones with data plans were provided. Third, there could have been increased study participation within the SmartPhone group due to lower rates of depression. Interactions of cognitive impairment or depression with the treatment effect of each intervention were not completed, due to the small sample size in this pilot study.
Finally, our study evaluated short-term outcomes, assessment of the long term effects of a SmartPhone based intervention on weight loss and post-stroke depression is a goal for future studies."

21) Generalisability (external validity, applicability) of the trial findings

NPT: External validity of the trial findings according to the intervention, comparators, patients, and care providers or centers involved in the trial

21-i) Generalizability to other populations

Generalizability to other populations: In particular, discuss generalizability to a general Internet population, outside of a RCT setting, and general patient population, including applicability of the study results for other organizations

	1	2	3	4	5	
subitem not at all important	○	○	○	●	○	essential

Does your paper address subitem 21-i?

Copy and paste relevant sections from the manuscript (include quotes in quotation marks "like this" to indicate direct quotes from your manuscript), or elaborate on this item by providing additional information not in the ms, or briefly explain why the item is not applicable/relevant for your study

"This study provided useful data on designing subsequent weight management trials for obese minority stroke survivors. Both the SmartPhone and the food journal intervention resulted in weight loss. Data from this study suggests that post-stroke depression improves with SmartPhone based engagement. Future minority research studies that include treatment of post-stroke depression and cognitive rehabilitation may positively influence intervention efficacy."

21-ii) Discuss if there were elements in the RCT that would be different in a routine application setting

Discuss if there were elements in the RCT that would be different in a routine application setting (e.g., prompts/reminders, more human involvement, training sessions or other co-interventions) and what impact the omission of these elements could have on use, adoption, or outcomes if the intervention is applied outside of a RCT setting.

	1	2	3	4	5	
subitem not at all important	○	○	●	○	○	essential

Does your paper address subitem 21-ii?

Copy and paste relevant sections from the manuscript (include quotes in quotation marks "like this" to indicate direct quotes from your manuscript), or elaborate on this item by providing additional information not in the ms, or briefly explain why the item is not applicable/relevant for your study

We used a PROBE design, which mimics a clinical setting more closely than a RCT.

OTHER INFORMATION

23) Registration number and name of trial registry

Does your paper address CONSORT subitem 23? *

Copy and paste relevant sections from the manuscript (include quotes in quotation marks "like this" to indicate direct quotes from your manuscript), or elaborate on this item by providing additional information not in the ms, or briefly explain why the item is not applicable/relevant for your study

TRIAL REGISTRATION: ClinicalTrials.gov NCT02531074

24) Where the full trial protocol can be accessed, if available

Does your paper address CONSORT subitem 24? *

Cite a Multimedia Appendix, other reference, or copy and paste relevant sections from the manuscript (include quotes in quotation marks "like this" to indicate direct quotes from your manuscript), or elaborate on this item by providing additional information not in the ms, or briefly explain why the item is not applicable/relevant for your study

Ifejika NL, Noser EA, Grotta JC, Savitz SI. Swipe out Stroke: Feasibility and efficacy of using a smart-phone based mobile application to improve compliance with weight loss in obese minority stroke patients and their carers. Int J Stroke. 2016;11:593-603.

25) Sources of funding and other support (such as supply of drugs), role of funders

Does your paper address CONSORT subitem 25? *

Copy and paste relevant sections from the manuscript (include quotes in quotation marks "like this" to indicate direct quotes from your manuscript), or elaborate on this item by providing additional information not in the ms, or briefly explain why the item is not applicable/relevant for your study

"Dr. Ifejika's current work: UT Southwestern / Texas Health Resources Clinical Scholar Award (#4)
Dr. Ifejika's and Dr. Cai's previous work: Center for Clinical and Translational Sciences at the McGovern Medical School at UTHealth, funded by NIH/NCATS Clinical and Translational Award UL1 TR000371 and KL2 TR000370. The content is solely the responsibility of the authors and does not necessarily represent the official views of the National Center for Research Resources or the NIH.
Dr. Ifejika's preliminary work: NIH/NINDS Diversity Supplement to P50 NS 044227, the University of Texas Specialized Program of Translational Research in Acute Stroke (SPOTRIAS)."

X27) Conflicts of Interest (not a CONSORT item)

X27-i) State the relation of the study team towards the system being evaluated

In addition to the usual declaration of interests (financial or otherwise), also state the relation of the study team towards the system being evaluated, i.e., state if the authors/evaluators are distinct from or identical with the developers/sponsors of the intervention.

	1	2	3	4	5	
subitem not at all important	○	○	●	○	○	essential

Does your paper address subitem X27-i?

Copy and paste relevant sections from the manuscript (include quotes in quotation marks "like this" to indicate direct quotes from your manuscript), or elaborate on this item by providing additional information not in the ms, or briefly explain why the item is not applicable/relevant for your study

"The Lose It! Mobile application is freely available. Ascend for Lose It! Was provided to study participants at no cost. Neither the study participants nor any members of the research team are owners or employees of Internet companies that market the services described in the manuscript. "

About the CONSORT EHEALTH checklist

As a result of using this checklist, did you make changes in your manuscript? *

- (●) yes, major changes
- ( ) yes, minor changes
- ( ) no

What were the most important changes you made as a result of using this checklist?

Time spent on each intervention, more descriptors in the Figures and Intervention section. Refining the abstract and manuscript text.

How much time did you spend on going through the checklist INCLUDING making changes in your manuscript *

Six hours

As a result of using this checklist, do you think your manuscript has improved? *

- (x) yes
- ( ) no
- ( ) Other:

Would you like to become involved in the CONSORT EHEALTH group?

This would involve for example becoming involved in participating in a workshop and writing an "Explanation and Elaboration" document

- ( ) yes
- ( ) no
- (x) Other: Would consider it

Any other comments or questions on CONSORT EHEALTH

Your answer

STOP - Save this form as PDF before you click submit

To generate a record that you filled in this form, we recommend to generate a PDF of this page (on a Mac, simply select "print" and then select "print as PDF") before you submit it.

When you submit your (revised) paper to JMIR, please upload the PDF as supplementary file.

Don't worry if some text in the textboxes is cut off, as we still have the complete information in our database. Thank you!

Final step: Click submit !

Click submit so we have your answers in our database!

Submit

Never submit passwords through Google Forms.

This content is neither created nor endorsed by Google. Report Abuse - Terms of Service - Privacy Policy

Google Forms
